# KIAA1199 deficiency enhances skeletal stem cell differentiation to osteoblasts and promotes bone regeneration

**DOI:** 10.1038/s41467-023-37651-1

**Published:** 2023-04-10

**Authors:** Li Chen, Kaikai Shi, Nicholas Ditzel, Weimin Qiu, Florence Figeac, Louise Himmelstrup Dreyer Nielsen, Michaela Tencerova, Justyna Magdalena Kowal, Ming Ding, Christina Møller Andreasen, Thomas Levin Andersen, Moustapha Kassem

**Affiliations:** 1 Department of Endocrinology and Metabolism, Endocrine Research Laboratory (KMEB), Odense University Hospital & University of Southern Denmark, Odense, Denmark; 2 Department of Orthopaedic Surgery and Traumatology, Odense University Hospital & University of Southern Denmark, Odense, Denmark; 3 Institute of Pathology, University of Southern Denmark, Odense, Denmark; 4 Department of Cellular and Molecular Medicine (ICMM), Faculty of Health Sciences, University of Copenhagen, Copenhagen, Denmark; 5 Present Address: Dept. of Pathology and Physiopathology, Guangxi Key Laboratory of Tumor Immunology and Microenvironmental Regulation, Guilin Medical University, Guilin, Guangxi China

**Keywords:** Bone, Mesenchymal stem cells

## Abstract

Upon transplantation, skeletal stem cells (also known as bone marrow stromal or mesenchymal stem cells) can regulate bone regeneration by producing secreted factors. Here, we identify KIAA1199 as a bone marrow stromal cell-secreted factor in vitro and in vivo. KIAA1199 plasma levels of patients positively correlate with osteoporotic fracture risk and expression levels of KIAA1199 in patient bone marrow stromal cells negatively correlates with their osteogenic differentiation potential. KIAA1199-deficient bone marrow stromal cells exhibit enhanced osteoblast differentiation in vitro and ectopic bone formation in vivo. Consistently, KIAA1199 knockout mice display increased bone mass and biomechanical strength, as well as an increased bone formation rate. They also exhibit accelerated healing of surgically generated bone defects and are protected from ovariectomy-induced bone loss. Mechanistically, KIAA1199 regulates osteogenesis by inhibiting the production of osteopontin by osteoblasts, via integrin-mediated AKT and ERK-MAPK intracellular signaling. Thus, KIAA1199 is a regulator of osteoblast differentiation and bone regeneration and could be targeted for the treatment or management of low bone mass conditions.

## Abstract (web summary)

The levels of a factor secreted by bone marrow stromal cells, KIAA1199, associate with osteoporotic fracture risk. Here, the authors show that KIAA1199 deficiency can lead to enhanced bone formation, accelerated bone healing, and protects from ovariectomy-induced bone loss.

## Introduction

Clinical trials employing bone marrow skeletal (also known as stromal or mesenchymal) stem cells (BMSC) for enhancing bone tissue regeneration, have revealed that the observed positive effects are majorly mediated by secreted factors^1^. Interestingly, the secretome of BMSC contains a large number of factors with unknown functions within bone biology, and with a possible therapeutic potential^2–4^. Also, these factors seem to be present at a biologically significant concentrations in the circulation, suggesting a systemic regulatory effect^5^. Another common feature of these novel factors is that their expression and secretion are regulated during BMSC lineage-specific differentiation, and in turn, they regulate at osteogenic differentiation, bone development, and bone mass^6–8^.

We have previously described the composition of the secretome of human BMSC (hBMSC) during osteoblastic differentiation as revealed by quantitative mass-spectrometry-based proteomic analysis^9^. Among the identified factors is KIAA1199, which is a 150 KDa protein encoded by a gene located on chromosome 15q25.1, has a G8 domain, contains 8 conserved glycine residues, and consists of 5 beta-chain pairs and 1 alpha helix^10^. Interestingly, mutation of the KIAA1199 causes human familial non-syndromic hearing loss^11^. The expression levels of KIAA1199 are increased in a number of cancer types^12–15^, and have been reported as a poor prognostic factor in patients with breast, colon cancer, and gastric cancer^12,14,16,17^, probably due to increased risk for metastasis^15,18–20^. Also, tissue expression of KIAA1199 is increased in the synovial tissues in patients with osteoarthritis (OA) and rheumatoid arthritis (RA)^21–24^. In addition, KIAA1199 binds and degrades hyaluronic acid (HA)^21,25^, a mechanism suggested to regulate endochondral ossification^26^. We have also reported that KIAA1199 regulates the motility and migration of hBMSC through changes in p38 MAPK kinase and Wnt signaling pathways^27^. According to these diverse biological functions, KIAA1199 is also called a ‘cell migration-inducing and hyaluronan-binding protein’ (CEMIP).

In the present study, we investigated the biological role of KIAA1199 in hBMSC biology in vitro and its impact on bone formation in vivo. Our results demonstrate that KIAA1199 is a regulator of osteoblastic differentiation and bone mass, bone strength, bone fracture healing, as well as bone loss following estrogen deficiency. Moreover, the KIAA1199 expression levels of BMSC that obtained from patients are negatively correlated with their osteogenic potentials, and the plasma levels of KIAA1199 exhibit a positive correlation with the clinical estimate of fracture risk.

## Results

### KIAA1199 is highly expressed in bone, BMSC and in the circulation

**Fig. 1:**
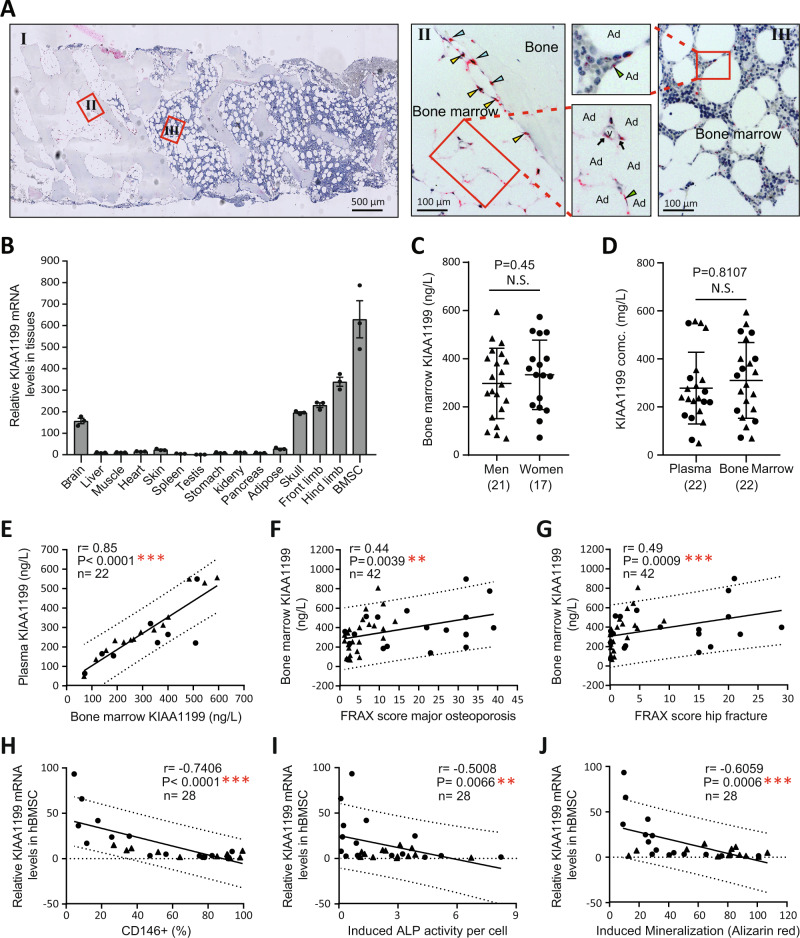
The plasma levels of KIAA1199 are positively correlated with osteoporotic fracture risk, but negatively correlated with osteogenic potentials of bone marrow stromal stem cells in patients. **A** In situ hybridization (ISH) analysis of KIAA1199 mRNA expression in human iliac crest bone biopsies showing positive staining (red dots) in osteoprogenitor cells (yellow arrowheads), bone-forming osteoblasts (blue arrowheads), bone marrow cells (black arrows) lining bone marrow adipocytes, *n* = 3 independent experiments. **B** The expression levels of KIAA1199 mRNA were examined by qRT-PCR in different mouse tissues, *n* ≥ 3 biologically independent samples. **C**–**E** Paired bone marrow supernatant fluids and peripheral blood plasma were collected in a group of patients admitted to the hospital with bone fractures, the levels of KIAA1199 in bone marrow were compared between men and women (**C**) and the paired KIAA1199 levels in bone marrow and peripheral plasma were compared (**D**) and the correlation was analyzed (**E**), *n* = 22. **F**, **G** Bone marrow KIAA1199 levels were correlated to FRAX score as an index for risk of osteoporotic (**F**) and hip fractures (**G**), *n* = 42. **H**–**J** KIAA1199 expression was measured in cultured human bone marrow stromal cells (hBMSC) obtained from the patients. The number of osteoblastic CD146 + cells was determined by flow cytometry analysis (**H**), and the cells were induced to osteoblastic differentiation that was evaluated by alkaline phosphatase (ALP) activity on day 7 (**I**) and formation of mineralized matrix visualized by Alizarin Red staining at day 14 (**J**). The correlation between the KIAA1199 mRNA levels and CD146 + cells percentage, or induced ALP activity and Alizarin Red staining eluted absorbance were analyzed in hBMSC, *n* = 42. Data is presented as mean ± SD, the comparison between two groups were analyzed by two-tailed unpaired Student’s t test (**C**–**D**), the correlation statistical analyses between variables were performed using the Spearman two-tailed correlation test (**E**–**J**). *P < 0.05, **P < 0.01 and ***P < 0.001. Source data are provided as a Source Data file.

We have previously identified KIAA1199 as a protein secreted by cultured hBMSC^9,28^. To identify its in vivo cellular production, we performed in situ hybridization on human iliac crest bone biopsies. KIAA1199 is expressed in the ‘reversal cells’ and the ‘canopy cells’ i.e., osteoprogenitor cells present along the bone formation sites (Fig. 1A). In addition, KIAA1199 is expressed in a group of marrow cells surrounding marrow adipocytes (Fig. 1A). KIAA1199 expression was absent from osteocytes and osteoclasts. Gene expression analysis performed on murine tissues showed that KIAA1199 is expressed at high levels in brain, and in bones (skull and limbs), as well as in cultured BMSC (Fig. 1B). Considering the relative tissue volume, bone tissue is probably the main source of KIAA1199 production in the body. Also, KIAA1199 is detected in peripheral blood and bone marrow plasma by ELISA and its levels were highly correlated in the two compartments, the levels were also similar in men and women (Fig. 1C–E & Supplementary Fig. S1A, B). To test for clinical relevance, plasma levels of KIAA1199 were determined in a cohort of patients admitted to the hospital with bone fractures as described in a previous publication from our group^29^. We found that KIAA1199 levels in bone marrow plasma positively correlate with osteoporotic fracture risk as determined by FRAX score^30^ (Fig. 1F, G & Supplementary Fig. S1C–D). The data suggest that KIAA1199 is a potential biomarker to be included in the assessment of osteoporotic fracture risk. Additionally, we found that KIAA1199 expression levels in cultured hBMSC obtained from the same cohort^29^ negatively correlates with the number of CD146 + osteoprogenitor cells, and also with in vitro induced cellular alkaline phosphatase (ALP) activity and mineralization capacity as visualized by Alizarin Red staining (Fig. 1H–J & Supplementary Fig. S2A–C).

### KIAA1199 inhibits osteoblast differentiation and bone formation of BMSC

**Fig. 2:**
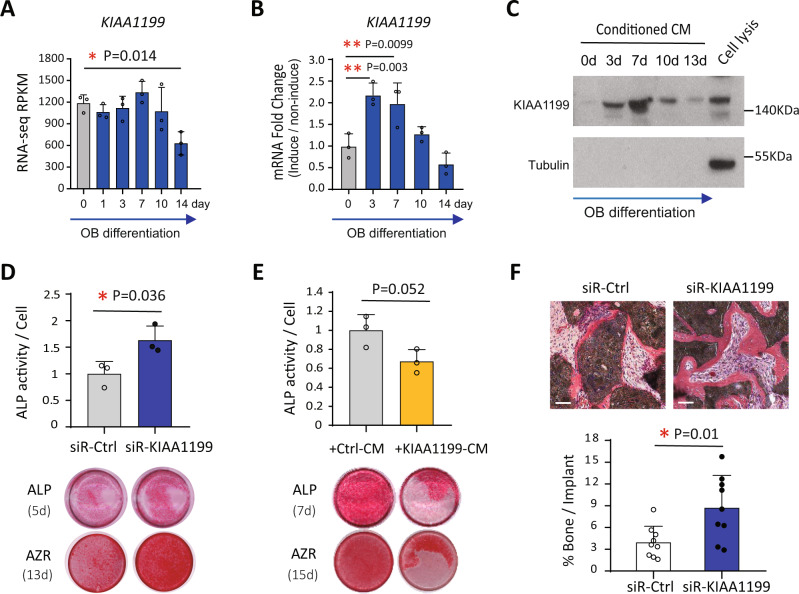
KIAA1199 is a regulator of osteoblast differentiation of human bone marrow stromal stem cells (hBMSC) in vitro and bone formation in vivo. **A**–**C** The expression of KIAA1199 in hBMSC was quantified by RNA-seq (**A**), real time PCR (**B**) and Western blot analysis in conditioned medium (**C**) at day (d) 0–14 during in vitro osteoblastic (OB) differentiation. The beta-tubulin was used to check the conditioned medium without any cell pellets contamination. Data was collected from at least three independent experiments; the representative results are shown here. **D** hBMSC were transfected with specific siRNAs targeting KIAA1199 (siR-KIAA1199) or non-target control siRNA (siR-Ctrl) and induced to osteoblast (OB) differentiation, *n* ≥ 3 independent experiments; **E** Conditioned medium (CM) from KIAA1199-overexpressing cells (+ KIAA1199-CM) and control vector cells were added to hBMSC during induction of OB differentiation of hBMSC. ALP activity (Day 7) and ALP staining (Day 5–7) and Alizarin Red staining (AZR) staining (Day 12–14) were measured during OB differentiation, *n* ≥ 3 independent experiments. **F** siR-Ctrl and siR-KIAA1199 transfected hBMSC have loaded into hydroxyapatite/tricalcium phosphate (HA/TCP) scaffolds and implanted subcutaneously in immune-deficient mice for 8 weeks. Newly formed bone tissues (red) were quantified to the whole implant area, *n* = 10 biologically independent samples. Scale bar **(F)** = 100 µm. Data are expressed as means ± SD. Statistical difference was determined by one-way ANOVA with Dunnett’s multiple test (**A**, **B**) or two-tailed unpaired Student’s t-test (**D–F**). *P < 0.05, **P < 0.01. Source data are provided as a Source Data file.

To determine the regulatory role of KIAA1199 in osteoblast differentiation in vitro, we first observed that KIAA1199 levels increase during the matrix formation stage (day 3–7) and decreased in the matrix mineralization stage (day 14) of osteoblast differentiation, as determined by RNA-Seq, real-time PCR and Western blot analysis (Fig. 2A–C & Supplementary Fig. S3). siRNA-mediated knockdown of KIAA1199 in hBMSC (siR-KIAA1199) strongly inhibited cellular expression of KIAA1199 as shown by low levels of gene expression (Fig. 6D) and low protein levels (Fig. 6H). siR-KIAA1199 cells exhibited enhanced OB differentiation evidenced by higher ALP activity, ALP staining intensity and mineralized matrix formation, compared to cells transfected with non-target control siRNA (siR-Ctrl) (Fig. 2D). On the other hand, conditioned medium (CM) from cells overexpressing KIAA1199 ( + KIAA1199 CM) inhibited OB differentiation (Fig. 2E). Moreover, siR-KIAA1199 cells loaded in hydroxyapatite/tricalcium phosphate scaffolds and implanted subcutaneously in immune-deficient mice, formed more in vivo ectopic bone tissue compared to siR-Ctrl hBMSC (Fig. 2F). The results demonstrate that KIAA1199 is a negative regulator for OB differentiation and bone formation in vitro and in vivo.

### KIAA1199 knockout (KO) mice exhibit higher bone mass and improved bone strength

To investigate the physiological role of KIAA1199 in bone, we generated KIAA1199 KO mice by CRISPR-Cas9 technology. As shown in Supplementary Fig. S4, Sanger sequencing confirmed that homozygous KO mice have a consistent 14 bp deletion in KIAA1199 gene (Supplementary Fig. S4A–C), and KIAA1199 protein was undetectable in brain tissue where it is highly expressed (Supplementary Fig. S4D). To exclude the presence of off-target effects, 10 potential off-target sites in the genome were tested (listed in Supplementary Table 1) and did not reveal signs of cleavage as demonstrated by T7E1 assay and Sanger sequencing analysis of the PCR products (primers listed in Supplementary Table 2).

**Fig. 3:**
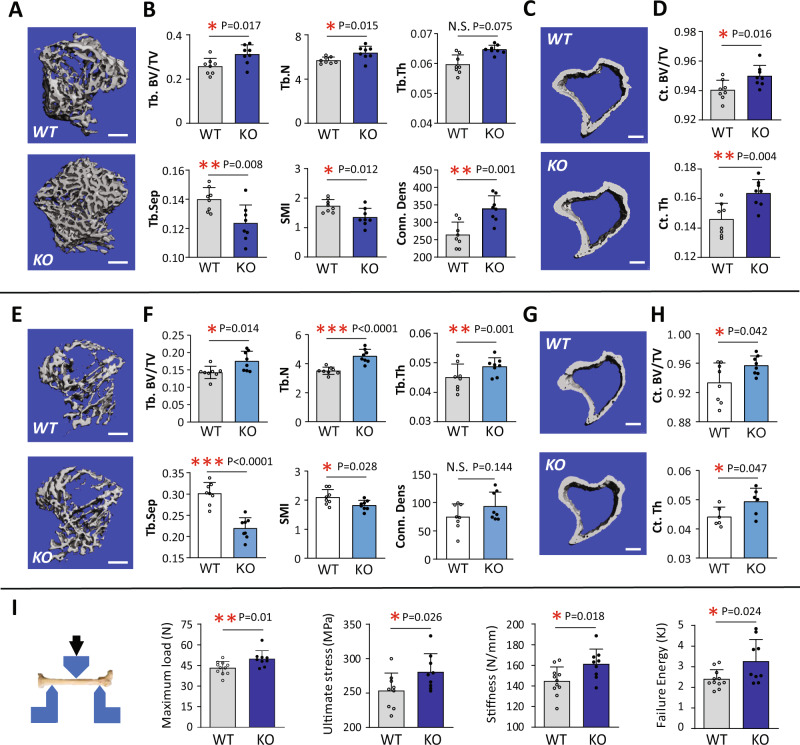
KIAA1199 KO mice exhibit increased bone mass and bone strength. **A**–**H** Bone mass was determined by µCT-scanning of trabecular bone (**A**, **B**, **E**, **F**) and cortical bone (**C**, **D**, **G**, **H**) on proximal tibia of 8 weeks male (**A**–**D**) and female (**E**–**H**) KIAA1199 knockout (KO) and corresponding wild-type (WT) mice. Photomicrographs of representative µCT-3D scan are shown in (**A**, **C**, **E**, **G**), and quantitative data analysis are shown (**B**, **D**, **F**, **H**). Trabecular bone volume per tissue volume (Tb. BV/TV), trabecular thickness (Tb. Th., mm), trabecular number (Tb. N., /mm^3^), trabecular connectivity density (Conn-dens., /mm3), trabecular structure model index (SMI), trabecular separation (Tb.Sp., µm), cortical (Ct) BV/TV, Ct thickness (Ct. Th., mm), *n* = 8. Scale bar (**A**, **C**, **E**, **G**) = 400 µm. **I** Three-point bending test was measured on femur of KO and WT male mice and quantitated by maximum load (N), ultimate stress (MPa), bone stiffness (N/mm) and failure energy (KJ), *n* (WT) = 11, *n* (KO) = 9. At least two sets animal’s experiments were detected and confirmed for each test, the results from one set experiment were presented. Data is pressed as means ± SD, statistical difference was determined by two-tailed unpaired Student’s t-test between two groups. *P < 0.05. **P < 0.01 and ***P < 0.001. Source data are provided as a Source Data file.

MicroCT (µCT) scanning of the proximal tibia of KIAA1199 male KO mice revealed increased trabecular bone volume / total volume (BV/TV), trabecular number (Tb. N.), trabecular thickness (Tb. Th.) and trabecular connectivity density (Conn-dens.), while decreased at trabecular structure model index (Tb. SMI.) and trabecular separation (Tb. Sp.), compared to corresponding wild-type (WT) mice (Fig. 3A, B). Similarly, the cortical bone parameters: BV/TV and cortical thickness were increased (Fig. 3C, D). Similar phenotype of trabecular and cortical bone mass was detected in female KO mice (Fig. 3E–H). Moreover, these changes in bone phenotype were confirmed at different ages in both male and female mice (Supplementary Fig. S5). The increase in bone mass in KIAA1199 KO mice, was associated with improved bone mechanical strength determined by 3-point bending and significantly increased maximum load, ultimate stress, stiffness and failure energy of bone (Fig. 3I).

### KIAA1199 KO mice exhibit accelerated bone fracture healing and are protected from ovariectomy-induced bone loss

**Fig. 4:**
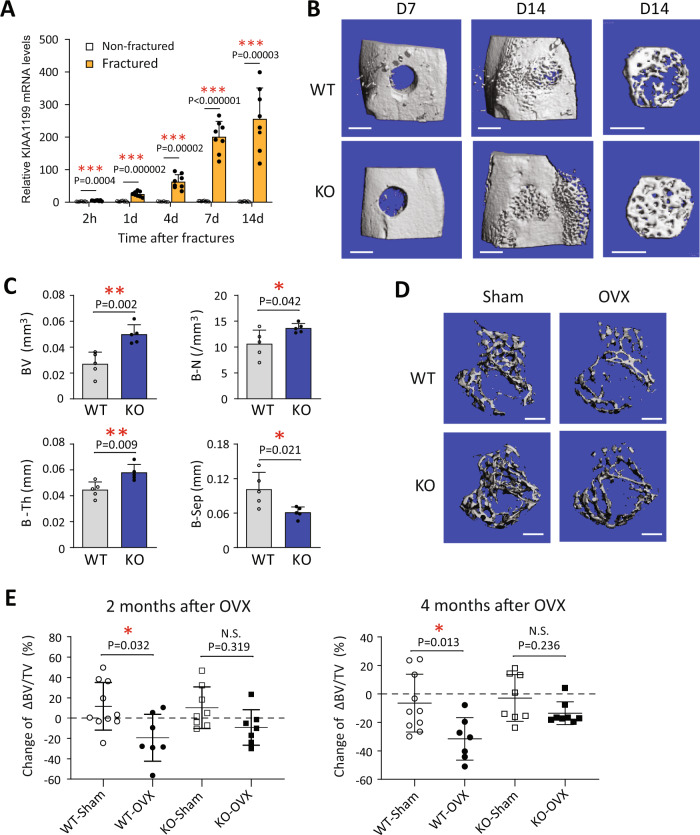
KIAA1199 KO mice exhibit accelerated bone regeneration and protection from ovariectomy-induced bone loss. **A** Gene expressions of KIAA1199 in mouse bone tissues at the site of tibial monocortical defect during 14 days bone regeneration, as measured by qRT-PCR, *n* = 8 biologically independent samples / group. **B**, **C** Bone regeneration in monocortical tibial defect in male KIAA1199 KO mice and WT mice. Representative 3D reconstruction of tibial defect by µCT at Day (D) 7 and 14 following the defect surgery (**B**). Bone volume (BV), trabecular bone number (B-N), trabecular bone thickness (B-Th) and trabecular bone separation (B-Sep) were quantitated on Day 14 (**C**), *n* = 5/group. **D**, **E** KIAA1199 KO and WT female mice (8-week-old) were subjected to sham-operation or ovariectomy (OVX), and bone mass of tibiae was quantified by µCT scanning on 2 and 4 months after OVX. The representative 3D reconstructed images on 4 months (**D**) and quantified changes in trabecular bone volume per tissue volume change (BV/TV) (**E**) are presented, *n* (WT-sham) = 10, *n* (WT-OVX) = 7, *n* (KO-Sham) = 8, *n* (KO-OVX) = 8. Scale bar (**B**, **D**) = 400 µm, at least two sets animal’s experiments were detected and confirmed for each test, the results from one set experiment were presented. data is presented as means ± SD, and statistical difference was determined by two-tailed unpaired Student’s t-test (**A**, **C**) or one-way ANOVA with Tukey’s multiple comparisons (**E**). *P < 0.05 and **P < 0.01. Source data are provided as a Source Data file.

Since KIAA1199 regulates osteoblast differentiation and bone formation, we further examined its role in bone regeneration following skeletal injury in a murine monocortical tibial defect model^31^. We observed significantly increased KIAA1199 gene expression in bone tissues at the site of bone defects (Fig. 4A). MicroCT-scanning quantification of the regenerated bone tissue during healing showed that KIAA1199 KO mice exhibit significantly accelerated bone regeneration (Fig. 4B) as shown by increased newly formed bone tissue volume (BV), bone thickness (B-Th.), trabecular bone numbers (B-N.), decreased trabecular bone separation (B-Sep.) (Fig. 4C). In addition, compared to the corresponding wild-type controls, ovariectomized (OVX) KIAA1199 KO female mice were protected from OVX-mediated bone loss as shown by changes in bone mass 2 and 4 months following OVX (Fig. 4D, E & Supplementary Fig. S6). These results demonstrate that KIAA1199 plays a role in bone formation following bone injury and following estrogen deficiency.

### KIAA1199 deficiency enhances osteoblastic differentiation and bone formation

**Fig. 5:**
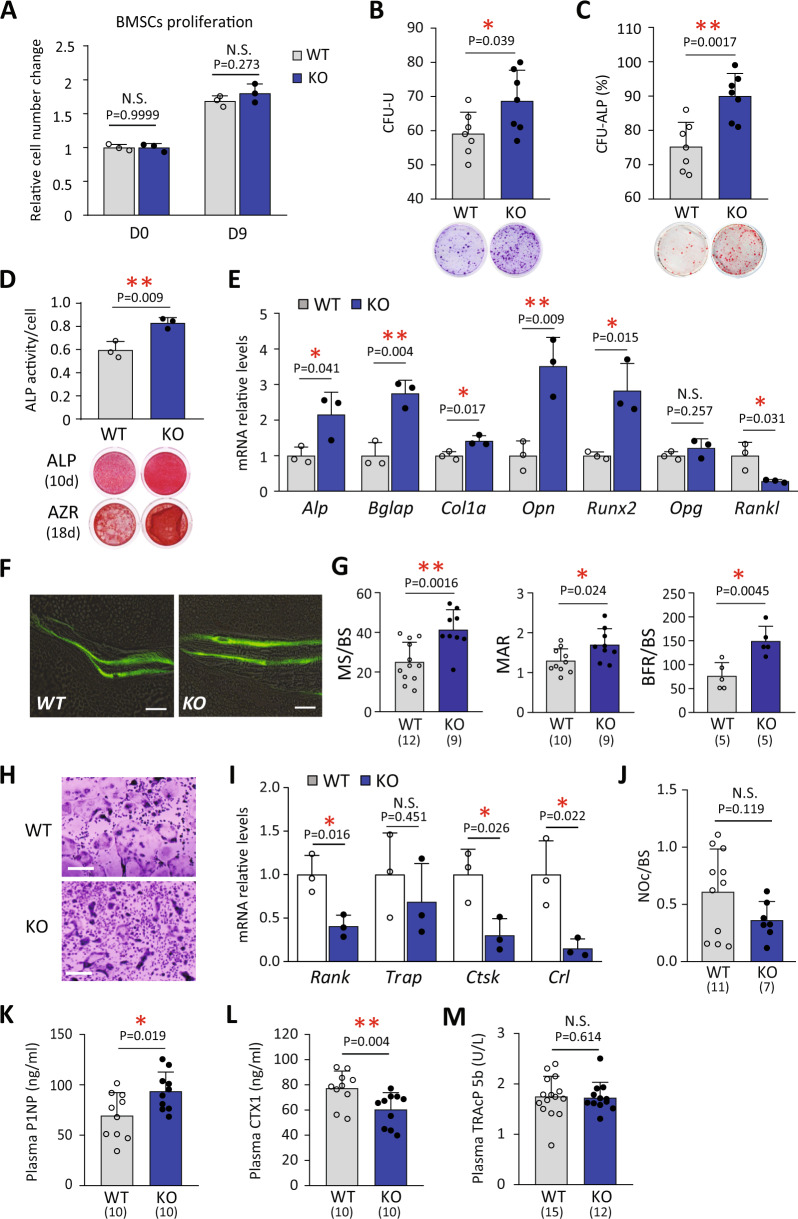
KIAA1199 KO increases osteogenic potential of bone marrow stromal stem cells, enhances the osteogenic differentiation but inhibits the osteoclast differentiation. **A** The changes of cell number of bone marrow stromal stem cells (mBMSC) from WT and KIAA1199 KO female mice were determined on day (D) 0 and 9, *n* = 4. **B**, **C** Colony-forming unit fibroblasts (CFU-Fs) were counted (**B**), CFU-AL*P* + colonies on day 7 were presented as a percent of CFU-Fs, *n* = 6. **D**, **E** Alkaline phosphatase (ALP) activity and ALP staining on day 10 and alizarin red (AZR) staining on day 18 during osteoblast (OB) differentiation of mBMSC (**D**); Real-time PCR analysis of OB gene markers: *Alp, alkaline phosphatase, Bglap osteocalcin, Col1a, collagen type 1, Opn, osteopontin, Runx2, Runt-related transcription factor 2, Opg, osteoprotegerin, Rankl, Receptor activator of nuclear factor kappa-Β ligand* (**E**), *n* ≥ 3. **F**, **G** Dynamic bone histomorphometry were measured in proximal tibia. Mineralized surface per bone surface (MS/BS, %), mineral apposition rate (MAR, µm/d), bone-formation rate per bone surface (BFR/BS, µm^3^/µm^2^/year) were compared between KO and WT male mice, *n* values are as labeled. **H**, **I** Osteoclast differentiation of bone marrow cells from female KIAA1199 KO and WT mice. The formation of multinucleated OC was verified by positive staining for tartrate-resistant acid phosphatase (TRACP) on day 5 (**H**). The expressions of OC maker genes were analyzed by real time PCR: *Rank, tumor necrosis factor receptor superfamily member 11* *A, Trap, tartrate-resistant acid phosphatase type 5, Ctsk, cathepsin K, Crl, calcitonin receptor* (**I**), *n* ≥ 3. **J** Number of osteoclasts per bone surface (NOc/BS, /mm) by bone histomorphometry on mice tibias, *n* values are as labeled. **K**–**M** Plasma levels of procollagen type 1 N-terminal pro-peptide (P1NP) (**K**), C-telopeptide of type I collagen (CTX1) (**L**) and tartrate-resistant acid phosphatase 5b (TRAcP 5b) (**M**) in KIAA1199 KO and WT mice were measured by ELISA, n values are as labeled. Scale bar (**F**, **H**) = 100 µm, date is presented as mean ± SD, statistical difference between WT and KO was determined by two-tailed unpaired Student’s t-test. *P < 0.05 and **P < 0.01. Source data are provided as a Source Data file.

We further explored the cellular mechanisms for the increased bone mass observed in KIAA1199 KO mice. We did not detect significant changes in cell proliferation of KIAA1199 KO mBMSC (Fig. 5A) which is consistent with previously reported results obtained from KIAA1199 deficient hBMSC^28^. On the other hand, the number of colony‐forming units-fibroblasts (CFU‐Fs) (representing osteoprogenitor cells) and the percentage of ALP positive CFU (CFU‐AL*P* + ) were significantly increased in KIAA1199 KO cultures compared to WT controls (Fig. 5B, C). Also, cultured KIAA1199 KO mouse BMSC displayed an enhanced in vitro OB differentiation, assessed by increased ALP activity, mineralized matrix formation evidenced by Alizarin red (AZR) staining (Fig. 5D), and increased gene expressions of osteoblastic marker genes: *Alp, Bglap, Col1a, Opn, Runx2* (Fig. 5E). Consistent with the in vitro effects, dynamic histomorphometry revealed increased mineralized surface per bone surface (MS/BS), mineral appositional rate (MAR) and tissue level bone-formation rate (BFR/BS) in KIAA1199 KO mice, compared with WT mice (Fig. 5F, G). Since bone mass is dependent on the balance between osteoblastic (OB) bone formation and osteoclastic (OC) bone resorption, we performed a preliminary assessment of osteoclastic cells. KIAA1199 deficiency did not change the expression of Opg, but significantly inhibited Rankl expression in the osteoblastic cells (Fig. 5E). Moreover, as shown in Fig. 5H, I, KIAA1199 KO cells formed fewer tartrate-resistant acid phosphatase (TRAcP) positive multinucleated osteoclastic cells and exhibited decreased OC gene markers expression: *Rank, Trap, Ctsk,* and *Crl*. However, the number of osteoclastic cells per bone surface and the plasma TRAcP did not show significant changes in KIAA119 KO mice. (Fig. 5J, M). On the other hand, KIAA1199 KO mice have higher plasma levels of bone formation marker procollagen type 1 N-terminal propeptide (P1NP) (Fig. 5K), and decreased levels of bone resorption marker C-telopeptide of type I collagen (CTX1) (Fig. 5L). The results suggest the effects of KIAA1199 in bone are mainly mediated through osteoblastic cells with a secondary effect on osteoclastic cells.

### KIAA1199 regulates osteoblast (OB) differentiation through osteopontin (OPN) / integrin / Akt / Erk pathways

**Fig. 6:**
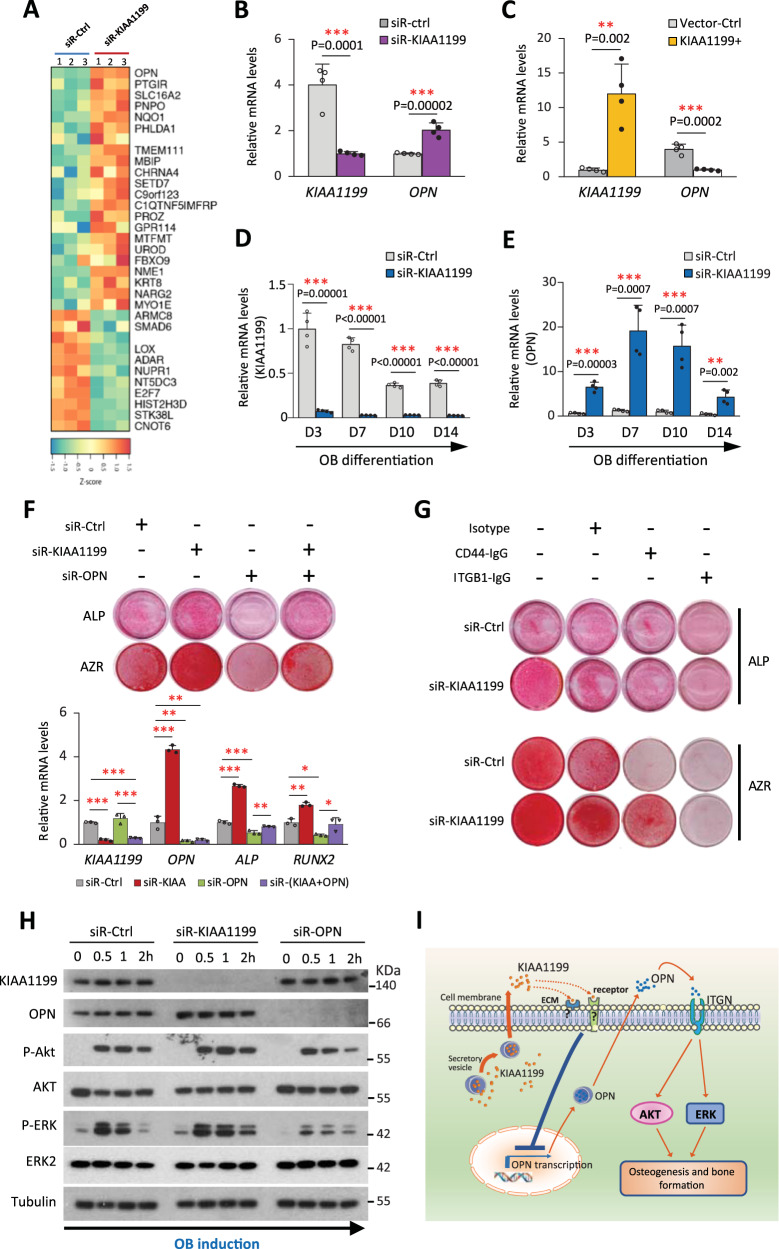
KIAA1199 regulates osteoblastic (OB) differentiation through OPN / integrin / AKT / ERK signaling. **A** Heat map of the most regulated genes identified by DNA microarray expression profiling in KIAA 1199 deficient human bone marrow stromal stem cells (hBMSC, transfected with specific siR-KIAA1199), compared to non-target siRNA transfected control hBMSC (siR-Ctrl), *n* = 3. **B**, **C** KIAA1199 and osteopontin (OPN) gene expression in siR-KIAA1199 and siR-Ctrl hBMSC (**B**); or in hBMSC overexpressing KIAA1199 by transfection with KIAA1199 plasmid (KIAA1199 + ) or control empty vector (Vector-Ctrl) (**C**). *n* = 4. **D**–**E** The expression of KIAA1199 (**D**) and OPN (**E**) were traced by qRT-PCR during OB differentiation in KIAA 1199 deficient hBMSC (siR-KIAA1199) and control hBMSC (siR-Ctrl) on Day (D) 3, 7, 10 and 14, *n* = 4. **F** hBMSC were transfected with specific siRNAs for KIAA1199 (siR-KIAA1199) or OPN (siR-OPN), or both, or non-target control (siR-Ctrl). ALP and Alizarin Red staining were performed at day 7 or 14 following OB induction. The expressions of KIAA1199, OPN, ALP and RUNX2 were detected on day 12, *n* = 3. **G** siR-KIAA1199 or siR-Ctrl hBMSC were treated with a blocking antibody against integrin β1 (ITGB1), or an isotype control, or blocking antibody against CD44 (each at 10 mg/ml) during OB differentiation. ALP and Alizarin red staining were performed on day 7 or 14. Gene expression of KIAA1199, OPN, ALP, RUNX2 were measured. **H** hBMSC transfected by siR-KIAA1199 or siR-OPN, or siR-Ctrl were starved in serum free medium for 6 hours, then added OB differentiation induction cocktail for 0 to 2 h. Proteins were subjected to Western blot analysis; beta-tubulin was used as the loading control. Data was collected from at least three independent experiments; the representative results are shown here. Data are expressed as means ± SD. Statistical difference was determined by one-way ANOVA. *P < 0.05, **P < 0.01 and ***P < 0.001. Source data are provided as a Source Data file. **I** A working model for the possible mechanism of KIAA1199 on regulating osteoblast differentiation by interaction with OPN / integrin.

To investigate the molecular mechanisms underlying KIAA1199 effects on OB differentiation, we performed global transcriptomic analysis on siR-KIAA1199 hBMSC and non-targeting siRNA controls by DNA microarray and identified osteopontin (OPN) as the most up-regulated gene (Fig. 6A) which was confirmed by real time qRT-PCR (Fig. 6B). On the other hand, overexpression of KIAA1199 in BMSC decreases OPN levels (Fig. 6C). Moreover, inhibition of KIAA1199 expression during the OB differentiation in BMSC (Fig. 6D), significantly up-regulated OPN gene expression (Fig. 6E). OPN is a non-collagenous extracellular matrix protein with regulatory role on bone formation. We detected that OPN mediates the observed effects of KIAA1199 as OPN deficiency induced by siRNA results in a significantly decreased OB differentiation shown by reduced ALP activity, mineralized matrix formation, as well as the expression of OB marker genes: ALP and RUNX2 (Fig. 6F & Supplementary Fig. S7). While combined knockdown of OPN and KIAA1199 blocked the enhanced OB differentiation observed in KIAA1199 deficient cells (Fig. 6F & Supplementary Fig. S7). Since OPN mediates its effects mainly through binding to its two receptors: integrin β1 or CD44^32,33^, we blocked integrin β1 or CD44 by a specific neutralizing antibody. Blocking CD44 or integrin β1 reduces OB differentiation of hBMSC, but only blocking integrin β1 reverses the enhanced OB differentiation observed in KIAA1199 deficient hBMSC (Fig. 6G). The results suggest that both integrin and CD44 signaling are important for OB differentiation of hBMSC, but only integrin β1 mediates the regulatory effects of KIAA1199-OPN on OB differentiation. To determine downstream signaling involved in KIAA1199-OPN interaction, we examined the major intracellular signaling pathways regulating OB differentiation. Inhibiting of KIAA1199 expression increased the activation of AKT and ERK phosphorylation (P-AKT, P-ERK), while inhibiting OPN expression led to the opposite effects (Fig. 6H), corroborating their opposite regulatory effects on OB differentiation of BMSC (Fig. 6F). Furthermore, KIAA1199 KO mice have increased plasma OPN levels in both males and females (Supplementary Fig. S8). The data suggest that KIAA1199 regulates the transcription and production of OPN, and modulating AKT and ERK signaling pathways known to regulate OB differentiation and bone formation. A working model based on these findings is illustrated in Fig. 6I.

## Discussion

Through global analysis of transcriptomics and proteomics of bone tissues and hBMSC, we have identified a number of bone non-canonical factors with a potential regulatory roles in bone biology^9^. In this study, we demonstrated that KIAA1199 is a secreted ‘osteokine’ from bone and BMSC that exerts regulatory effects on osteoblastic differentiation and bone formation through interaction with OPN / integrin signaling.

We observed high expression of KIAA1199 in bone tissues and in brain and with lower expression levels in other organs including liver and kidneys. Within the bone, KIAA1199 is expressed specifically in osteoprogenitor cells colonizing eroded surfaces, of what has been termed reversal cells and ‘canopy cells’^34^. Both cell populations defined by their physical location, are recruited to bone-forming surfaces as bone-forming cells and they also mediate coupling between bone resorption and bone formation^34^. These findings corroborate the findings of a previous study from our group that has revealed the ability of KIAA1199 to enhance BMSC cell migration and motility^28^.

We observed that KIAA1199 levels in bone marrow plasma and blood plasma are closely correlated suggesting equilibrium between bone/bone marrow compartment and peripheral blood. Also, this data suggest that bone tissue contributes significantly to KIAA1199 blood levels. KIAA1199 is highly expressed in bone, cartilage, stromal stem cells, and brain. Developing tissue-specific knockouts may provide information regarding the relative contribution of bone versus other tissues to circulation levels of KIAA1199. On the other hand, since KIAA1199 is a circulating factor, use of global knockout model is relevant since the whole body will be exposed to circulating KIAA1199. In this study, we found that the plasma KIAA1199 levels significantly correlate with the fracture risk, and the expression of KIAA1199 in human BMSC correlates with their osteogenic potentials. Moreover, we also observed that plasma KIAA1199 levels are higher in patients with osteoporosis compared to non-osteoporosis controls, in a population sample that included men and women (Supplementary Fig. S1, E). Further clinical studies with more samples which could exclude the sex and age bias are needed to determine the use of KIAA1199 as a biomarker in the assessment of osteoporotic fracture risk.

KIAA1199 deficient mice exhibit high bone mass, and enhanced bone formation in both males and females. The bone formed is of high biomechanical quality as it exhibited increased bone strength in ex vivo three-point bending tests. In addition to its effects on bone mass under steady-state conditions, KIAA1199 deficiency also enhances healing of surgically-generated bone defects and prevented excessive bone loss following ovariectomy. Both conditions are known to involve significant changes in the osteoprogentior cell populations^34^. These positive effects are possibly mediated by several cellular mechanisms identified in our study. First, increased number of osteoprogenitors cells as shown by the increased number of CFU-F and CFU-ALP^+^. Second, enhanced osteoblastic number and functions as shown by the upregulation of osteoblastic marker genes. Third, possibly reduced osteoclastic cell differentiation. Thus, KIAA1199 is a possible coupling factor that inhibits bone formation and enhances bone resorption. While the observed effects on bone formation have been consistent, the effects on osteoclastic bone resorption showed some variations. For example, plasma levels of CTX-1 were significantly reduced in KIAA1199 KO mice, but plasma TRAcP 5b levels and the number of osteoclasts/ bone surface measured by histomorphometry did not exhibit significant changes. Additional detailed studies are needed to identify possible regulatory mechanisms of KIAA1199 on osteoclastic bone resorption.

Another plausible mechanism for the increased bone formation by KIAA1199 deficiency is changes in extracellular matrix remodeling. KIAA1199 cleavages high molecular-weight (MW) hyaluronic acid (HA) leading to the degradation of HA^21^. Accumulation of high MW HA in bone microenvironment regulates the cellular activities of osteoblastic cells through a number of signaling pathways known to affect bone cell functions^35,36^. For example, in a previous study employing KIAA1199 deficient mouse, abnormalities in endochondral ossification and shortened adult long bones were caused by the accumulation of high MW HA in the lengthened hypertrophic zone of the growth plates^26^. In the current study, we identified OPN as a possible mediator of KIAA1199 effects on bone remodeling. OPN is a sialoprotein that is an important component of bone extracellular matrix. KIAA1199 deficiency up-regulates OPN expression in BMSC, and KIAA1199 knockout mice have increased plasma OPN levels. Interestingly, the bone phenotype of OPN-deficient mice is also the opposite of KIAA1199-deficient mice. For example, bone remodeling responses to mechanical stress were suppressed in OPN deficient mice^37^, and the mice exhibited an increased number of osteoclasts and enhanced bone resorption^38^.

Increased expression of KIAA1199 has been reported in a number of clinical conditions e.g. in patients with different cancer types^12,14,16,17^ and in patients with rheumatoid arthritis and osteoarthritis, both conditions are characterized by joint inflammation^21–24^. In our study, we found the global knockout of KIAA1199 in mice enhanced the bone density and stiffness. This change in vivo might have some association with other tissue functions regulated by KIAA1199, but the in vitro osteoblast differentiation of both human BMSC and mouse BMSC, and in vivo heterotopic bone formation proved that KIAA1199 has a direct and independent role on osteogenic differentiation and bone formation, but not a consequence effects from other tissues.

We report that the plasma levels of KIAA1199 are positively correlated with an increased risk for osteoporotic fracture and are higher in patients with osteoporosis. These observations corroborate and extend the in vivo findings in mice and suggest that KIAA1199 plays a similar role in human bone mass regeneration. The positive effects of KIAA1199 deficiency on bone mass in mice, encourage further clinical translational studies to target KIAA1199 as an approach to increase bone formation in patients with osteoporosis. Indeed, several studies have examined KIAA1199 as a therapeutic target for different diseases, e.g. inhibition of KIAA1199-mediated hyaluronan degradation to prevent excessive skin wrinkles^39,40^, or decreased KIAA1199 expression in joints to relieve synovial membrane inflammation in OA^41^. Developing small molecules targeting KIAA1199 or KIAA1199 specific neutralizing monoclonal antibodies will be useful in examining their therapeutic effects in bone diseases, such as non-healed bone fractures or osteoporosis.

## Methods

### Ethics statement

All animal works and protocols in the present study were approved by the Danish Animal Experiments Inspectorate (No. 2017-15-0201-01210). All the related measurements and surgeries on animals were performed in a rodent-dedicated animal center in Odense University Hospital and complied with all relevant ethical regulations for vertebrate animal research. During the living scanning of Dual-energy X-ray absorptiometry (DEXA) and Micro–computed tomography (µCT), mice were anesthetized with isoflurane (1% to 4%) in 100% oxygen during the measurements; for the surgeries of ectopic bone formation assay, monocortical tibia defect (MCTD) and ovariectomized (OVX), mice were anesthetized by intraperitoneal injection of ketamine (100 mg/kg body weight) and xylazine (5–10 mg/kg body weight). The induced anesthesia lasted enough for finishing all the surgeries. The mice under measurement or surgeries were monitored continually during and after the experiments for depth of anesthesia by response to pain, observation for movement, body temperature, and respiratory rate. Mice were operated on warmed operation beds, after the surgery, mice were kept warm and under-watch in a watch room for overnight before transferring to the regular feeding room. After surgery, analgesia was managed by necessary additional administration of with buprenorphine (0.1 mg/kg body weight subcutaneously every 6^th^ hour, or 1 mg/kg orally in Nutella® nut paste every 12^th^ hour, for 3 days). The pain and distress associated with the entire process were minimized. At the time of euthanasia, mice were deeply anesthetized with isoflurane (1.5% in oxygen; 1 L/min) before putting into carbon dioxide (CO_2_) chambers. All efforts to minimize animal suffering were made.

Human samples (blood plasma, bone marrow supernatant, and hBMSCs) were collected at the Department of Orthopedic and Traumatology, Odense University Hospital complies with the relevant ethical regulations. All subjects received oral and written project information and signed written consent, informed consent for generation, and subsequent use of the samples for the experiments in the present study were approved by the Scientific Ethics Committee of Southern Denmark (S-20160084, 2019).

### In situ hybridization

The mRNA expression of KIAA1199 in human bone was investigated by in situ hybridization using a modified version of the RNAScope2.5 high-definition procedure (310035, Advanced Cell Diagnostics [ACD], Hayward, CA, USA), as previously described^34^. The 3.5-µm-thick paraffin sections of decalcified 3-mm iliac crest needle biopsies from four human controls^42^ were pretreated, hybridized with 20 ZZ-pairs (Probe-Hs-CEMIP, 449811, ACD) targeting KIAA1199 (Region 1934-2896 of NM_001293298.1). The study was approved by the Danish Regional Committee on Biomedical Research Ethics (S-20070121).

### Cell culture

A telomerized hBMSC line (hBMSC-TERT) was established from bone marrow hBMSC cultured from a healthy male bone marrow through overexpression of human telomerase reverse transcriptase gene and has the ‘stemness’ characteristics^43^. Human bone marrow MSC (hBMSC) were cultured in basal culture medium with Minimum Essential Medium (MEM) with 10% fetal bovine serum (FBS) and 1% penicillin-streptomycin (P/S). Mouse bone marrow MSC (mBMSC) was cultured from tibia and femur bone marrow. Briefly, cells were filtered through 40 µm nylon mesh, and cultured in Minimum Essential Medium (MEM) with 20% FBS, 1 mM pyruvate, 1x Non-Essential Amino Acids and 1% P/S. Half of the medium was replaced every 3 days. At confluence, mBMSCs were harvested and passaged. Cells were incubated in 5% CO_2_ incubators at 37 °C. All reagents were purchased from Life Technologies (Taastrup, Denmark).

### Induction of osteoblast (OB) differentiation

hBMSC or mBMSC were cultured to reach confluence and the cells were then induced to osteoblast differentiation in osteogenic induction medium (OIM) containing standard growth medium supplemented with 10 nM dexamethasone, 0.2 mM L-ascorbic acid, 10 mM β-glycerophosphate, and 10 mM 1.25-vitamin-D3 (Sigma). Differentiation induction medium was changed every three days, the induction of differentiation lasted for 7-14 days according to the requirements for different measurements.

### Osteoclast culture and differentiation

Mouse bone marrow cells derived from less than 8 weeks old mice were plated in 96-well plates in osteoclastic medium containing a-MEM supplemented with 10% FBS, 100 U/mL of penicillin, 100 mg/mL of streptomycin, 25 ng/mL of recombinant human macrophage colony-stimulating factor (rhM-CSF; R&D Systems, Minneapolis, MN, USA), and 25 ng/ mL of recombinant human receptor activator of NF-kB (rhRANKL; Pepro-Tech, Rocky Hill, NJ, USA). The presence of osteoclastic cells was verified by staining for tartrate-resistant acid phosphatase (TRAcP) performed 5-7 days after culture, and TRAcP multinucleated (≥ 3) cells were scored as osteoclastic cells.

### Evaluation of osteoblast differentiation

Alkaline phosphatase (ALP) activity was performed in 96-well plate. Firstly, cell number (viable cells) was determined by adding the CellTiter-Blue solution (Promega, Madison, USA) for 2 hours and estimated by measuring the fluorescence at wavelength of 579Ex/584Em. Then the cells were then rinsed with TBS (20 mM Trizma base, 150 mM NaCl at pH = 7.5) and fixed in 3.7% formaldehyde-90% ethanol for 30 seconds at room temperature. A reaction mixture containing 50 mM NaHCO_3_, 1 mM MgCl_2_ (Sigma-Aldrich), and 1 mg/ml of p-nitrophenyl phosphate (Sigma-Aldrich) was added into each well and incubated at 37 °C for 20 minutes. The reaction was stopped by adding 50 μl of 3 M NaOH. Absorbance was determined at 405 nm in an ELISA Microplate Reader. ALP enzymatic activity was normalized to cell number.

ALP staining was performed on cultured cells by rinsing the cell once with phosphate-buffered saline (PBS) and fixation in acetone/citrate (1.5:1, V: V) buffer (pH 4.2) for 5 minutes at room temperature. The cells were incubated with buffer containing 0.2 mg/ml naphthol AS-TR phosphate (Sigma) for one hour, then washed with deionized water. Alizarin Red S staining was employed to trace the formed mineralized matrix. Cell was rinsed with PBS and fixed in 70% ethanol for 1 hour at −20 °C. The fixed cells were stained with 40 mM Alizarin Red S pH 4.2 (Sigma) for 10 minutes at room temperature and then washed with deionized water. The photomicrograph images were acquired using an invented phased-contrast microscope (Zeiss, Oberkochen, Germany). Alizarin Red staining was extracted by hexadecylpyridinium chloride (Sigma C9002) and quantitated at OD570nm.

### In vivo ectopic bone formation assay

hBMSC (5 × 10^5^ cells / sample) were loaded on 40 mg wet hydroxyapatite/tricalcium phosphate (HA/TCP) (Zimmer), incubated at 37 °C overnight, and implanted subcutaneously on the dorsal side of 8-week-old NOD.CB17-Prkdcscid/J mice (NOD/SCID) in sterilized animal rooms. Implants were removed after 8 weeks and fixed in 4% paraformaldehyde, decalcified in formic acid, and embedded in paraffin. Sections (4 μm) were cut and stained with H&E. Bone volume per total volume was quantified as previously described^27^.

### Quantitative Real-Time qPCR

RNA was isolated by TRIzol according to the manufacturer’s instructions (Thermo-Fisher Scientific, Roskilde, Denmark). The first strand complementary DNA was synthesized from 1 µg total RNA by Revert aid cDNA kit (Sigma, Copenhagen, Denmark). The PCR products were visualized in real-time using SYBR Green I Supermix (Bio-Rad) and an iCycle instrument (Bio-Rad) using standard curve protocols, normalized to the geometric mean of the reference genes. The quantitative data presented is an average of duplicate or triplicate per independent experiment. Primer sequences for the genes tested are shown in Supplementary Table 3.

### Cell transfection

The transfection in cells was performed at 70–90% confluence using Lipofectamine^TM^ 2000 (Life Technologies, Taastrup, Denmark) with plasmids or siRNAs according to the manufacturer’s recommendations. The siRNAs for specific genes (siR-KIAA1199: s32900, s32899, s32901; OPN: s13377, s13375, s13376) or control non-targeting siRNA (4404021, 4390846) were all purchased from Silencer® Selector siRNA library from Thermo-Fisher Scientific (Roskilde, Denmark). The KIAA1199 (Gene ID: NM_001293298.1) expressing plasmid construct was produced with pLVX-mCMV-ZsGreen vector (Biowit, Shenzhen, China). The conditioned medium (CM) from the KIAA1199 overexpression hBMSC and corresponding vector-transfected cells was performed as described before^28^.

### Microarray analysis

Human MSCs transfected by siRNA-Ctrl or siRNA-KIAA1199 separately. After 72 hours, hMSC-siR-Ctrl and hMSC-siR-KIAA1199 cells were harvested and RNAs were extracted from 3 biological replicates. Total RNA was isolated using TRIzol (Invitrogen) firstly, treated by DNase I (Sigma-Aldrich), then purified again by GenElute mammalian total RNA miniprep kit (Sigma-Aldrich). The quantification and quality analysis of RNA was analyzed by the 2100 Bioanalyzer (Agilent). One microgram of total RNA was labeled according to the GeneChip Whole Transcript (WT) Sense Target Labeling Assay as provided by the manufacturer (Affymetrix) and hybridized to Affymetrix Whole-Transcript Human Gene 1.0 ST Arrays (Affymetrix) overnight before scanning in an Affymetrix GCS 3000 7 G scanner. Normalization was performed using Expression Console (Affymetrix, USA) with the RMA gene core algorithm. Clustering analysis of gene expression levels was done with Gene Cluster 2.11 (rana.lbl.gov / EisenSoftware.htm) using Pearson’s correlation and complete linkage and Java TreeView (ver. 1.1.3) for visualization of the heat map.

### Western blot analysis

The cells were washed in cold PBS and lysed in RIPA buffer (Thermo-Fisher Scientific, Roskilde, Denmark) supplemented with protease inhibitors (Roche, Hvidovre, Denmark) for more than 30 minutes. Samples were centrifuged at 12,000 x *g*, 4 °C for 10 minutes. Protein concentrations were determined with a BCA kit (Thermo-Fisher Scientific, Roskilde, Denmark), and equal amounts (30 µg) of proteins were loaded on a polyacrylamide gel (Thermo-Fisher Scientific, Roskilde, Denmark). Blotted nitrocellulose membranes were incubated overnight with primary antibody (1:1000 diluted) at 4 °C and were developed after 1 h incubation with secondary anti-rabbit or anti-mouse horseradish peroxidase-conjugated antibody (1:2000 diluted, #sc2004, #sc2005, Santa Cruz Biotechnology, USA) using an ECL Western blotting kit (GE Healthcare, USA) and Kodak films. Antibody for KIAA1199 (#21129-1-AP) was purchased from ProteinTech, Manchester, UK. Antibody for osteopontin (#ab69498, clone53) was from Abcam, Cambridge, UK, Antibody for alpha-tubulin (#T9026, clone DM1A) was from Sigma, Søborg, Denmark, and other antibodies used in the study (phos-AKT, #4051 clone587F11, AKT: #9272, phos-ERK: #4695, clone137F5, ERK: #9101) were purchased from Cell Signaling Technology (Herlev, Denmark). The full scans of the most important blots were attached in the Source Data file that provided with this paper.

### Generation of KIAA1199^-/-^ mice by CRISPR/Cas9 technology

The mouse KIAA1199 gRNA was designed (http://crispr.mit.edu), and the gRNA in the 4^th^ exon of mouse KIAA1199 genome (TACATCCTGATTGATGA CGG) was selected and inserted into the pSPCas9(BB)−2A-GFP (PX458) vector which was a gift from Feng Zhang (Addgene, Plasmid#48138)^44^. The constructed plasmid was confirmed by sequencing (Eurofins Scientific, Galton, Denmark), and delivered into mouse ES cells (Transgenic Core Facility, DanStem Centre, Copenhagen University, Copenhagen, Denmark). GFP-positive ES cells were sorted by FACS and expanded as single-cell clones. ES cell clones were screened by T7E1 mismatch cleavage assay and Sanger Sequencing, the proper gene-edited ES cell candidates were chosen to generate chimeric mice. After crossing with wild-type C57BL/6 J female mouse, KIAA1199^+/-^ mice were selected and inter-crossed to obtain homozygous KIAA1199 knockout (KIAA1199^-/-^, KO) mice. Speed-Congenic analysis was performed to obtain mice on pure C57BL/6 J background (Taconic Biosciences, Silkborg, Denmark).

Mice were fed ad libitum normal chow diet with 6% fat, 30% protein, 63% carbohydrate, 7.7% sucrose (Altromin, Lage, Germany, #132003). Animals were bred and housed under standard conditions (21 °C, 55% relative humidity) on a 12-hour light / dark cycle.

### T7E1 Assay and Sanger sequencing for genotyping

Tail biopsies for genotyping were taken from mice after weaning; DNA was extracted by DNeasy Blood & Tissue Kit (Qiagen, Germany). DNA samples were kept at 4 °C for several weeks or at –20 °C for long-term storage. Primers for T7E1 were designed (Forward: 5’ CTGTGCTTTGGGTTCACG 3’; Reverse: 5’ GCTAGACCTGGGTAGAATT 3’) to amplify 942 bp on either side of the KIAA1199 gRNA target genome by PCR production. PCR productions were denatured by heating at 95 °C for 5 min in a thermocycler (Biometra, Göttingen, Germany) and then formed the heteroduplexes by slowly cooling to 23 °C. T7 endonuclease I digestion was performed at 37 °C for 30 min using 5 U of T7 endonuclease I (New England BioLabs, Bionordika Denmark A/S, Herlev, Denmark) on 5 µL of unpurified PCR products in a reaction volume of 10 µL. The reactions were used for electrophoresis in 2% agarose gel and photographed. Two-round T7E1 mismatch cleavage assay was used to select KIAA1199^-/-^ mice. WT mice showed only one band. KIAA1199^-/-^ mice showed one band on 1^th^ round of T7E1 assay and two bands on 2^th^ round of T7E1 assay after mixing WT DNA to each mouse DNA sample. Furthermore, PCR productions were purified and sent to Eurofins Genomics (Eurofins Scientific, Galton, Denmark) for further DNA sequence analysis.

### Dual-energy X-ray absorptiometry (DEXA) and Micro–computed tomography (µCT) scanning

Mice total bone mineral density (BMD) was determined by dual-energy X-ray absorptiometry (DEXA) with a Lunar PIXImus2® densitometer (Version 1.44, Lunar Corporation, Madison, WI, USA). The tibias or femurs of mice were scanned with a microcomputed tomography (µCT) system (vivaCT40, Scanco Medical, Brüttisellen, Switzerland) by X-ray at 55 KVp volts for trabecular bone at a resolution of 10.5 µm/slice, the trabecular region started at 0.26 mm from the proximal growth plate and extended for 0.52 mm in the tibias. Cortical bones at mid-diaphysis of the tibia were scanned at 70 KV energy and 114 µA intensity. Bone volume (BV), bone volume fraction (BV/TV), trabecular number (Tb. N.), trabecular thickness (Tb. Th.), trabecular space (Tb. Sp.), and cortical thickness (Ct. Th.) were evaluated from 3D reconstruction of cubic images^45–47^.

### Monocortical tibia defect (MCTD) and ovariectomized (OVX) osteoporosis model

Male mice under anesthesia went surgery to create a monocortical tibia defect in the right leg of the animals^48^. Briefly, the lateral aspect of the right tibia was exposed, and a dental drill was used to obtain a monocortical osseous hole (0.8 mm diameter) on the anterior surface of the tibia crest. A saline solution was used to remove bone dust and fragments from the defect. The anterior tibial muscle was reset into its anatomical position and skin layers were sutured with 5-0 absorbable suture. The bone healing was traced by µCT on day 7 and 14 after the surgery.

At 8 weeks of age, female mice were ovariectomized (OVX) and followed anesthetized. A dorsal skin incision, approximately 1 cm long was made above the upper lumbar spine and gently moved laterally to enable access to both ovaries by blunt dissection of the muscle layer. The ovarian blood supply was electro-coagulated before the connection between the fallopian tube and the uterine horn was cut and the ovary was removed. The incision was then sutured with 3 single catgut stitches. Sham-operated (Sham) mice were used as controls. After 8 and 16 weeks after OVX, all groups were subjected to µCT for checking bone microstructure and densities.

### Bone biomechanical tests

Biomechanical testing of bone micro-hardness on femur was conducted as described previously^49^. Briefly, prior to mechanical test, micro-CT scanning was performed to measure the cortical thickness and diameters of each femoral cortical bone as inputs for calculating mechanical properties. Three-point bending test of bone specimen was performed on an 858 Bionix MTS hydraulic material testing system (MTS Systems Co., Minneapolis, Minnesota, USA), using a 1 KN load cell with a strain of 0.006. The two lower supports had a distance of 8.5 mm, and the upper loading point was at the middle of the cortical bone, i.e. 4.25 mm from each lower support. During test, the load-deformation curve was recorded and converted to stress-strain curve. The mechanical properties, such as maximal load was recorded, and ultimate stress (strength), ultimate strain, Young’s modulus (stiffness), and failure energy (fracture toughness) were calculated.

### Measurements on clinical cells and plasma samples from patients

Peripheral blood plasma, bone marrow supernatant fluid samples, and hBMSC were collected from the lower extremities of forty-two adult donors (including 24 males and 18 females in age between 18 and 97 years) undergoing routine orthopedic surgeries at the Department of Orthopedic and Traumatology, Odense University Hospital. Among patients, there were 26 individuals diagnosed with osteoporosis and 16 non-osteoporotic patients. Informed consent for the generation and subsequent use of the samples in the study were approved by the Scientific Ethics Committee of Southern Denmark (project ID S-20160084, 2019). The blood and bone marrow fluids were centrifuged, the supernatants were collected and stored at −80 °C till measurements. The hBMSC were cultured and subjected to qRT-PCR and induced OB differentiation, quantitated the ALP activity, and mineralization of differentiation was performed as described^29^.

### Enzyme-linked immunosorbent assay (ELISA)

Mouse fasting plasma were harvested before sacrifice, and human peripheral blood plasma and bone marrow supernatant fluids were collected from patients. Quantitative determinations of different factors were measured by the corresponding ELISA kits or activity assay kits (hKIAA1199: from Antibody Online, #ABIN457025; mOPN: from Abcam, # ab100734; mP1NP, mCTX-1, mTRAcP 5b: from Immunodiagnostic Systems, # AC-33F1, # AC-06F1, # SB-TR103) following the manufacture manuals.

### Statistical analysis

All the data were collected from at least two independent experiments and presented as the mean and standard deviation (SD). Normality test was applied to all data to test data distribution. Two tailed student’s t-test (a parametric or non-parametric) was used to assess differences between two groups’ comparisons according to the data distribution. One-way ANOVA was used for more than two groups’ comparisons with one condition, ANOVA analysis was followed by post hoc test for multiple comparisons. The correlation statistical analyses between variables were performed using the Spearman two-tailed correlation test (r = Spearman correlation coefficient). Analysis of correlation was performed using GraphPad Prism 7.1 software. The number of independent donors (n) in each correlation analysis is described in the results section and in each figure. Statistical significance was considered when *P* < 0.05.

### Reporting summary

Further information on research design is available in the Nature Portfolio Reporting Summary linked to this article.

## Supplementary information

Supplementary Information

Reporting Summary

## Data Availability

The data and materials that support the findings of this study are available from the corresponding author upon request. Microarray data (accession #GSE216863) have been deposited in NCBI’s Gene Expression Omnibus (GEO) public database (https://www.ncbi.nlm.nih.gov/geo/query/acc.cgi?acc=GSE216863). Source data are provided with this paper.

## Source data

Source Data
