# Supplementary material for: KIAA1199 deficiency enhances skeletal stem cell differentiation to osteoblasts and promotes bone regeneration
Source: Nat Commun. 2023 Apr 10;14:2016. doi: 10.1038/s41467-023-37651-1 (PMC10086002; doi:10.1038/s41467-023-37651-1)
Supplement: Supplementary file 1 — Supplementary Information [file 41467_2023_37651_MOESM1_ESM.pdf]

Title: KIAA1199 deficiency enhances osteoblast differentiation of skeletal stem cells and bone regeneration

Supplementary information contents:

1. Supplementary Figures: 1-8
2. Supplementary Tables: 1-3

Fig. S1

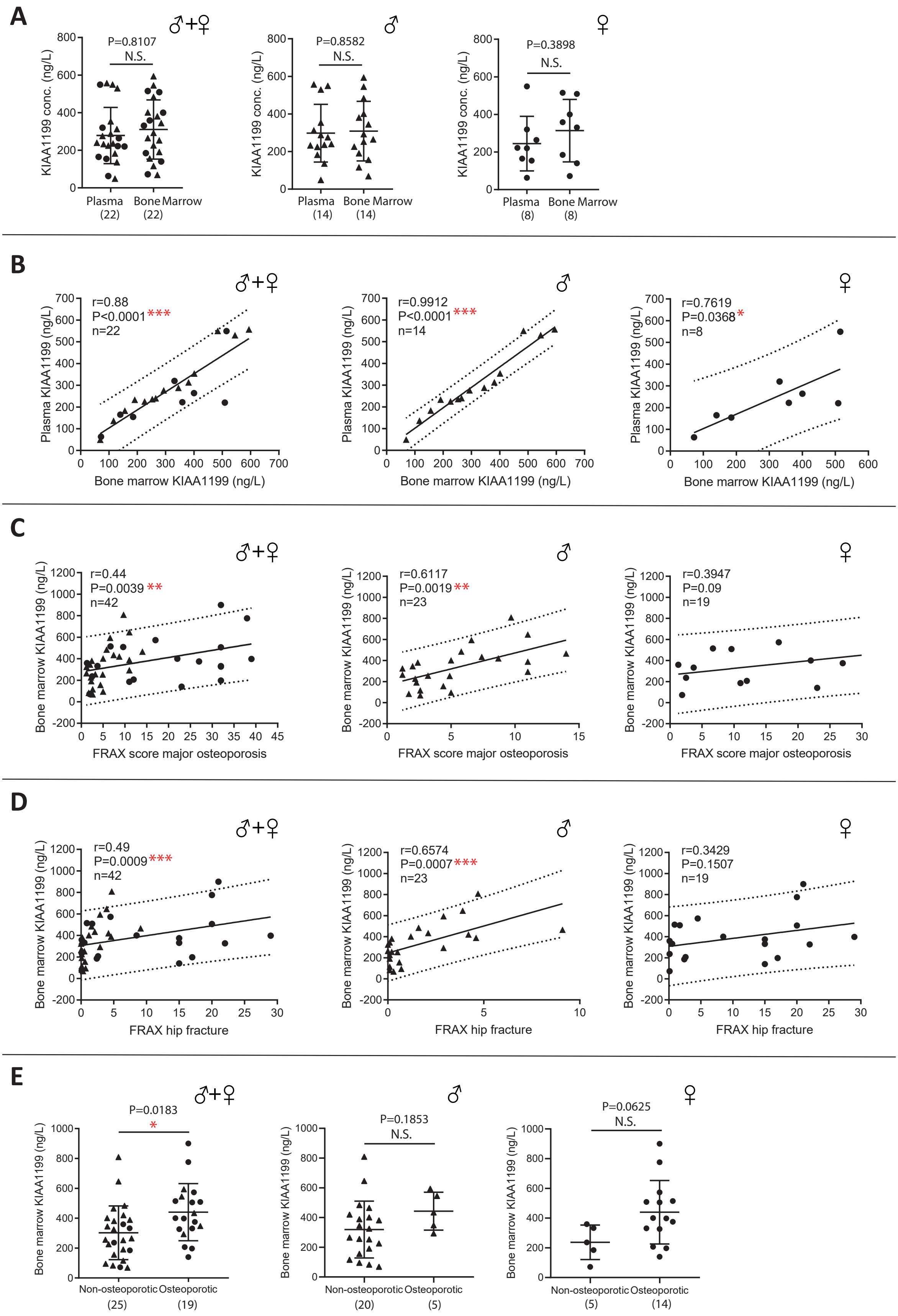
A
♂+♀
P=0.8107
N.S.
KIAA1199 conc. (ng/L)
Plasma (22)
Bone Marrow (22)
♂
P=0.8582
N.S.
Plasma (14)
Bone Marrow (14)
♀
P=0.3898
N.S.
Plasma (8)
Bone Marrow (8)
B
♂+♀
r=0.88
P<0.0001***
n=22
Plasma KIAA1199 (ng/L)
Bone marrow KIAA1199 (ng/L)
♂
r=0.9912
P<0.0001***
n=14
♀
r=0.7619
P=0.0368*
n=8
C
♂+♀
r=0.44
P=0.0039**
n=42
Bone marrow KIAA1199 (ng/L)
FRAX score major osteoporosis
♂
r=0.6117
P=0.0019**
n=23
♀
r=0.3947
P=0.09
n=19
D
♂+♀
r=0.49
P=0.0009***
n=42
FRAX hip fracture
♂
r=0.6574
P=0.0007***
n=23
♀
r=0.3429
P=0.1507
n=19
E
♂+♀
P=0.0183
*
Non-osteoporotic (25)
Osteoporotic (19)
♂
P=0.1853
N.S.
Non-osteoporotic (20)
Osteoporotic (5)
♀
P=0.0625
N.S.
Non-osteoporotic (5)
Osteoporotic (14)

Fig. S1. Analysis of bone marrow KIAA1199 serum levels in the clinical patients.

(A) Bone marrow supernatant fluids and peripheral blood plasma were collected in a group of patients admitted to the hospital with bone fractures, the levels of KIAA1199 in bone marrow and plasma were determined by ELISA and compared in both genders (n = 22) or men (n = 14, ♂) or women only (n = 8, ♀). (B) The correlation between paired bone marrow KIAA1199 levels and peripheral blood plasma KIAA1199 from the same patient was analyzed in both genders (n = 22, ♂+♀) or men (n = 14, ♂) or women only (n = 8, ♀). (C) The correlation of bone marrow KIAA1199 levels to FRAX score as an index for risk of major osteoporotic fractures was analyzed in clinical patients in both genders (n = 22, ♂+♀) or men (n = 14, ♂) or women only (n = 8, ♀). (D) The correlation of bone marrow KIAA1199 levels to FRAX score as an index for risk of hip fractures was analyzed in clinical patients in both genders (n = 22, ♂+♀) or men (n = 14, ♂) or women only (n = 8, ♀). (E) The osteoporosis in patients was diagnosed with a dual-energy X-ray absorptiometry (DEXA) scan at hip and spine, then the bone mineral density (BMD) was calculated to get a T-score from the bone mass of an average healthy adult in their twenties. T-score less than -2.5 was defined as osteoporosis. The bone marrow KIAA1199 levels were compared in osteoporosis and non-osteoporosis patients in both genders (n = 22, ♂+♀) or men (n = 14, ♂) or women only (n = 8, ♀). Data is presented as mean ± SD, the comparison between two groups were analyzed by two-tailed unpaired Student's t test, the correlation statistical analyses between variables were performed using the Spearman two-tailed correlation test. N.S., non-significant, *$P < 0.05$, **$P < 0.01$, ***$P < 0.001$. Source data are provided as a Source Data file.

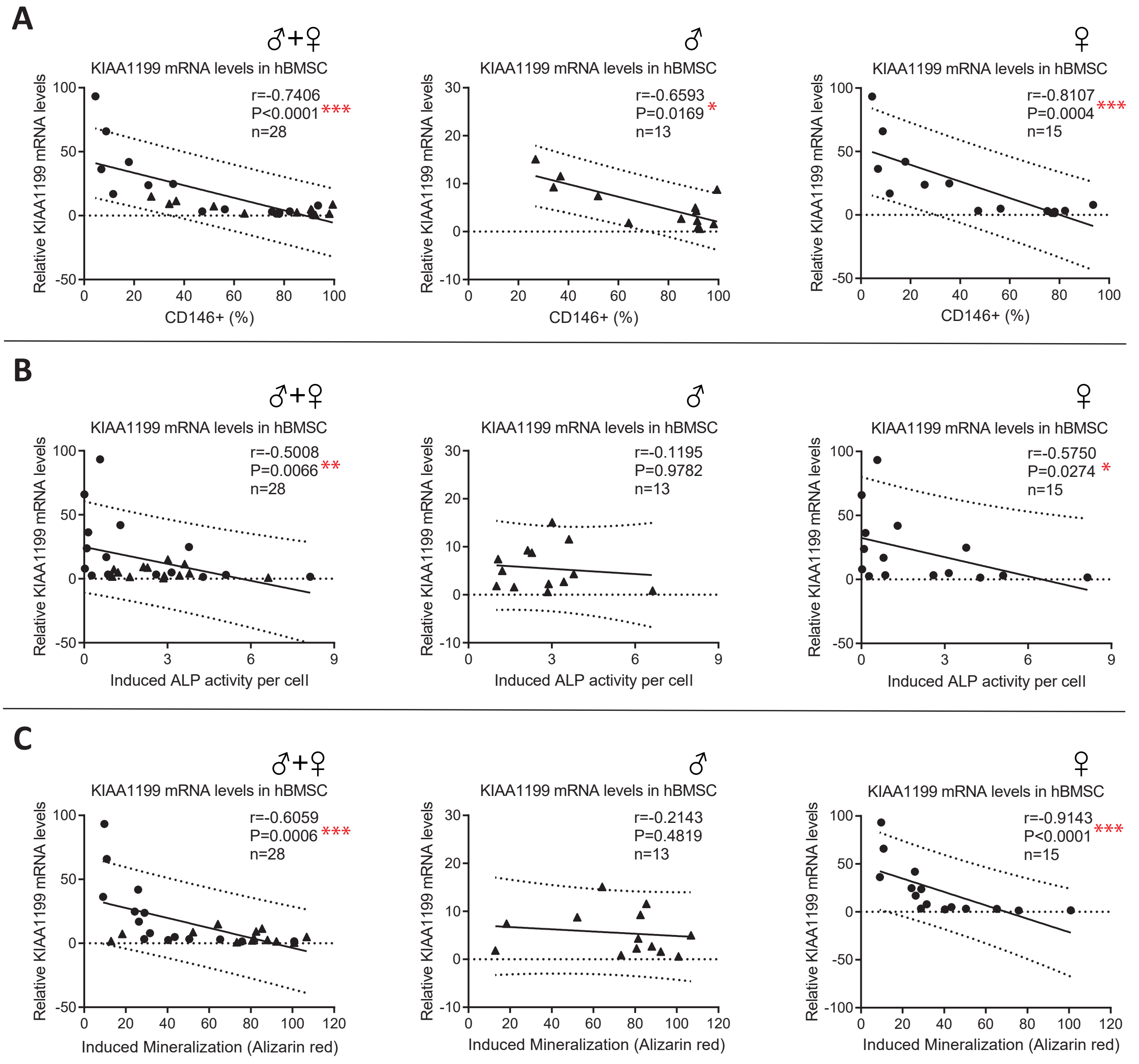

Fig. S2. Analysis of KIAA1199 expression levels in human bone marrow stromal cells (hBMSC) with the corresponding osteogenic potential of hBMSC in clinical patients.

KIAA1199 expression was measured in cultured human bone marrow stromal cells (hBMSC) obtained from the patients. The number of osteoblastic CD146+ cells was determined by flow cytometry analysis (A), and the cells were induced to osteoblastic differentiation that was evaluated by alkaline phosphatase (ALP) activity on day 7 (B) and formation of mineralized matrix visualized by Alizarin Red staining at day 14 (C). (A-C) The correlation between the KIAA1199 mRNA levels in hBMSCs and CD146+ cells percentage, ALP activity and Alizarin Red staining eluted absorbance were analyzed in both genders (n = 28, ♂+♀) or men (n = 13, ♂) or women only (n = 15, ♀). Data is presented as mean ± SD, the correlation statistical analyses between variables were performed using the Spearman two-tailed correlation test, *P < 0.05, **P < 0.01, ***P < 0.001. Source data are provided as a Source Data file.

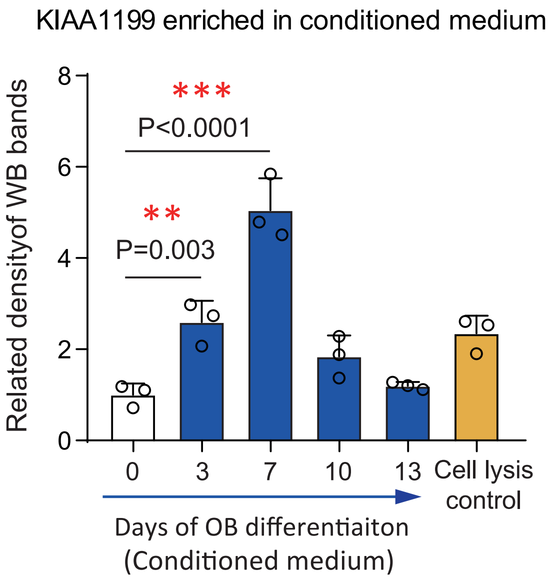

Fig. S3. Densitometric analysis of KIAA1199 secretion during osteoblast differentiation of hBMSC in Figure 2C. Integrated density of the Western blot that detected the KIAA1199 levels in concentrated (50:1) conditioned medium (CM) from human bone marrow stromal stem cells (hBMSCs) during the osteoblast (OB) differentiation on Day 0, 3, 7, 10, 13. Data is quantized by ImageJ software and presented as mean ± SD, n = 3. Statistical difference was determined by one-way ANOVA with Dunnett's multiple comparison, $^{**}P < 0.01$, $^{***}P < 0.001$. Source data are provided as a Source Data file.

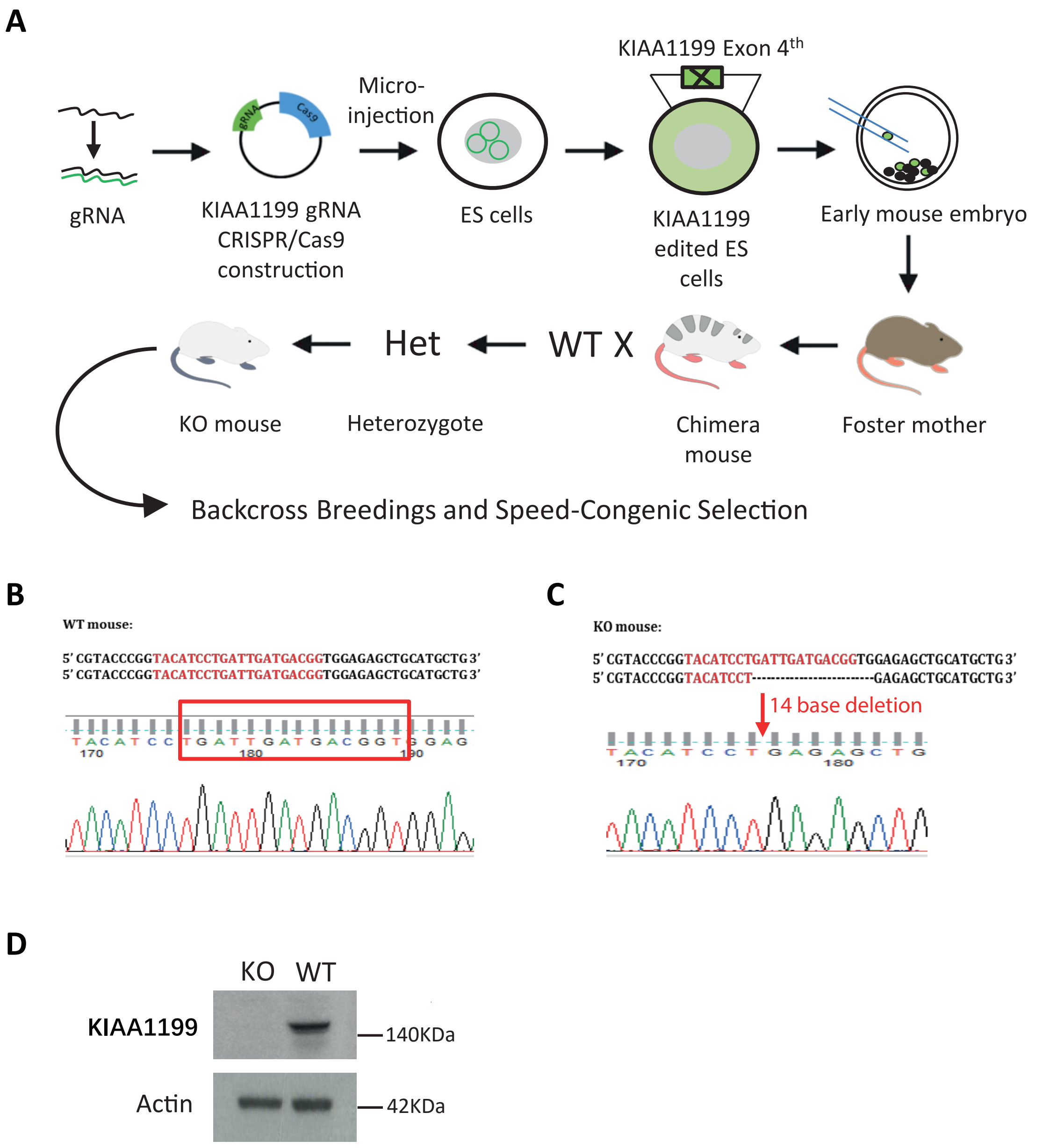

Fig. S4. Establishment of KIAA1199 full knock-out mice. (A) Diagram of Mouse KIAA1199 knock-out mice generation by CRISPR technology. (B-C) Sanger sequencing results showing sequence in KIAA1199 gRNA targeting region in KIAA1199 wild type (WT) and knockout (KO) mice. KO mice are deleted 14 bases in KIAA1199 gene compare to WT in the exon. (D) Mouse brain tissue protein (40 µg) was isolated from KIAA1199 knockout (KO) and wild type (WT) mice and subjected to Western blot analysis for protein expression of KIAA1199, beta-actin was used as loading control of protein samples. Data was collected from at least two independent experiments; the representative results are shown here. Source data are provided as a Source Data file.

Fig. S5

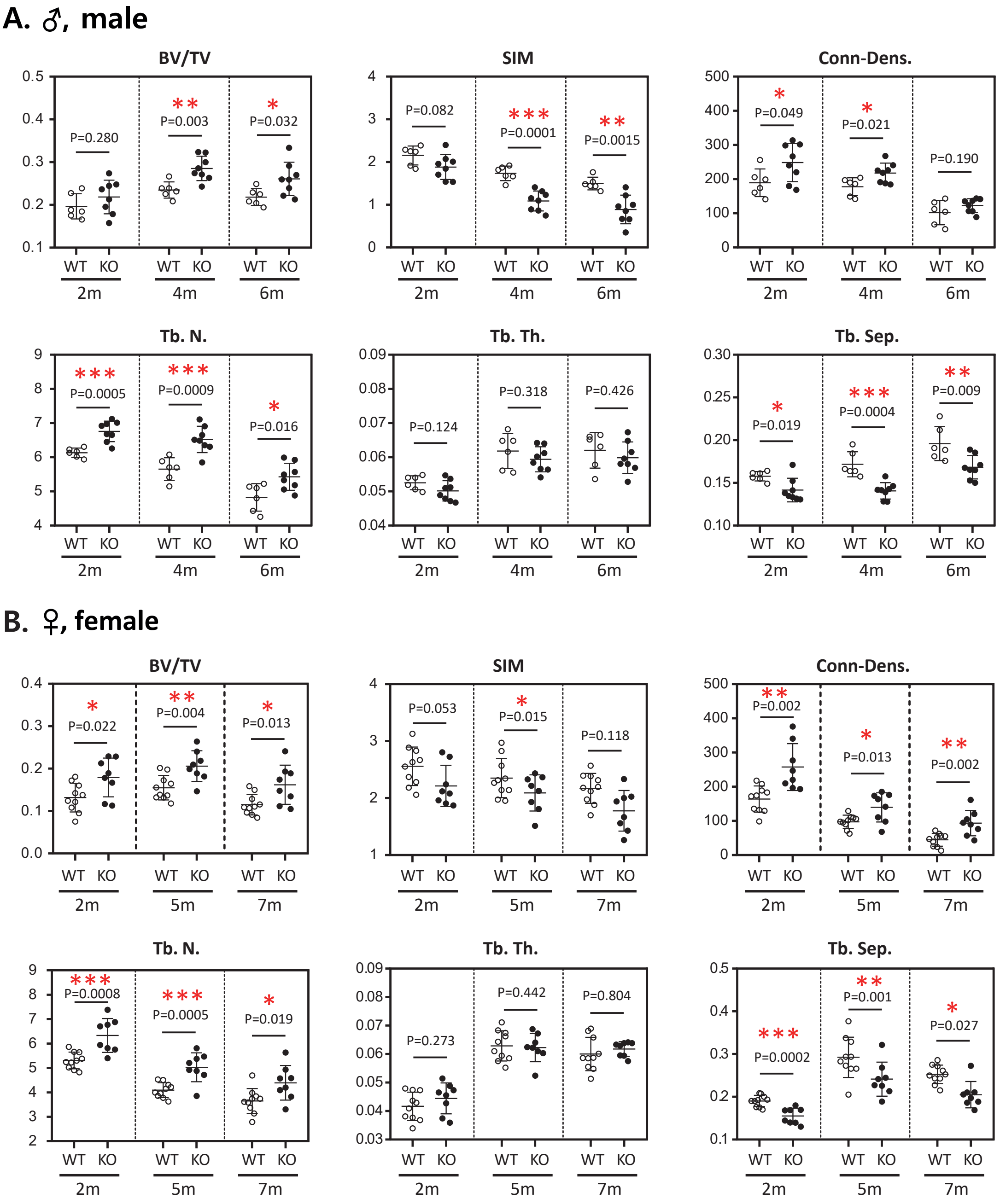

Fig. S5. Micro-computed tomography (μCT) scanning of bone mass of male (♂) and female (♀) KIAA1199 knockout (KO) and corresponding wild-type (WT) mice follow the age from 2-7 months. Bone mass was determined by μCT-scanning of trabecular bone on proximal tibia of KIAA1199 KO and WT mice in male (♂) at age of 2, 4, 6 months (m), and female (♀) at 2, 5, 7 months (m). Quantitative data analysis is shown as: trabecular bone volume per tissue volume (Tb. BV/TV), trabecular structure model index (SMI), connectivity density (Conn-dens., /mm^3), trabecular number (Tb. N., /mm^3), trabecular thickness (Tb. Th., mm), trabecular separation (Tb. Sep., μm), n (M-WT) = 6, n (M-KO) = 8, n (F-WT) = 10, n (F-KO) = 8. Data is presented as mean ± SD, the comparison between WT and KO at different age were analyzed by two-tailed unpaired Student's t test, *P < 0.05, **P < 0.01, ***P < 0.001. Source data are provided as a Source Data file.

Fig. S6

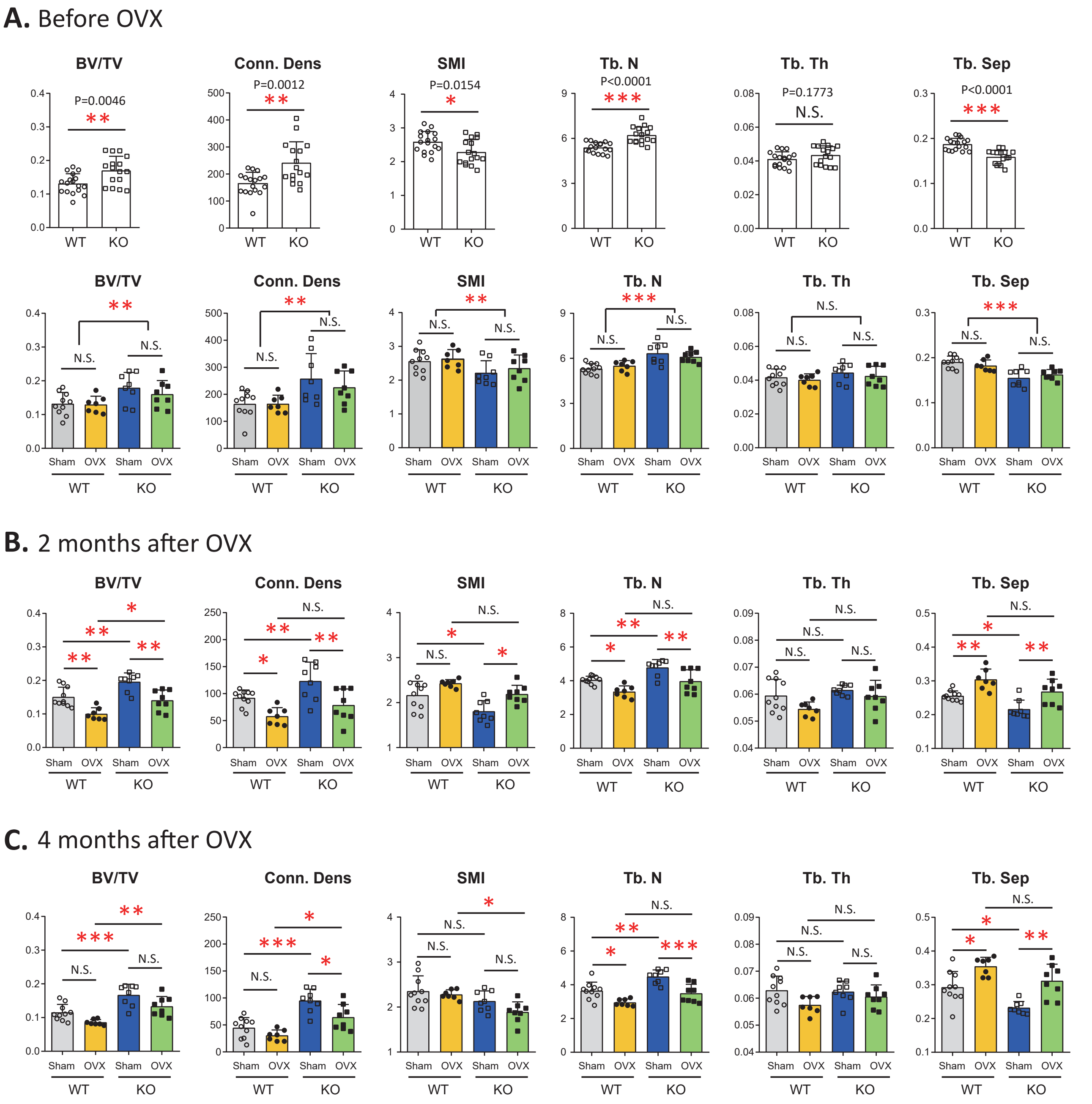

Fig. S6. Micro-computed tomography (μCT) scanning of bone mass of female (♀) KIAA1199 knockout (KO) and corresponding wild-type (WT) mice before and follow the sham-operation or ovariectomy (OVX) on 0- 2-4 months. KIAA1199 KO and WT female mice (8-week-old) were subjected to sham- or OVX-operation, and bone mass of tibiae was quantified by μCT scanning on 0, 2 and 4 months after OVX. Bone mass was determined by μCT-scanning of trabecular bone on proximal tibia of KIAA1199 KO and WT mice. Quantitative data analysis is shown as: trabecular bone volume per tissue volume (Tb. BV/TV), connectivity density (Conn-dens., /mm^3), trabecular structure model index (SMI), trabecular number (Tb. N., /mm^3), trabecular thickness (Tb. Th., mm), trabecular separation (Tb.Sep., μm). Data is expressed as means ± SD, n (WT-sham) = 10, n (WT-OVX) = 7, n (KO-Sham) = 8, n (KO-OVX) = 8. Statistical difference was determined by one-way ANOVA with Tukey's multiple comparisons for four groups or two-tailed unpaired Student's t-test between two groups, *P < 0.05, **P < 0.01 and ***P < 0.001. Source data are provided as a Source Data file.

Fig. S7

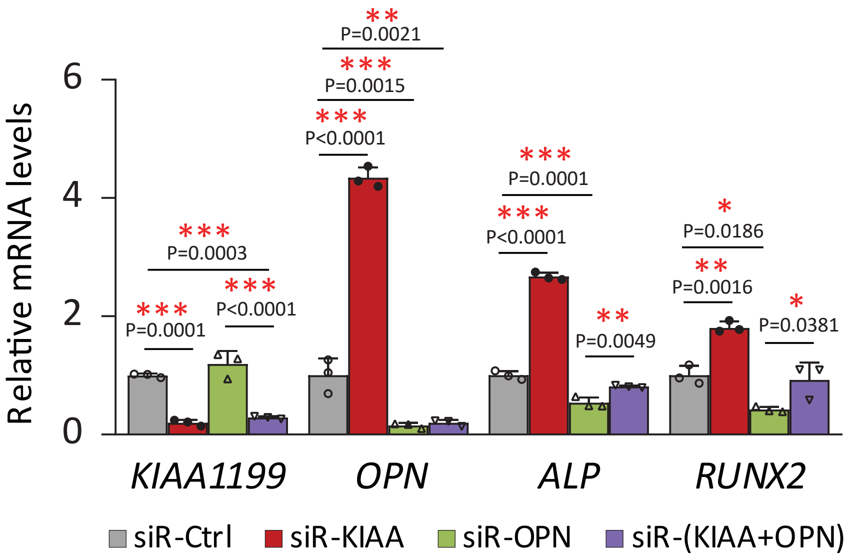

Fig. S7. Enlarged graph for Figure 6F with P values labeled. Human bone marrow stromal stem cell (hBMSC) were transfected with specific siRNAs for KIAA1199 (siR-KIAA1199) or OPN (siR-OPN), or both, or non-target controls (siR-Ctrl). Cells were induced to osteoblast (OB) differentiation for 14 days. The expressions of KIAA1199, OPN, ALP and RUNX2 were detected on day 12 by real time PCR, n = 3 independent experiments. Data is expressed as means ± SD. Statistical difference was determined by one-way ANOVA. *P < 0.05, **P < 0.01 and ***P < 0.001. Source data are provided as a Source Data file.

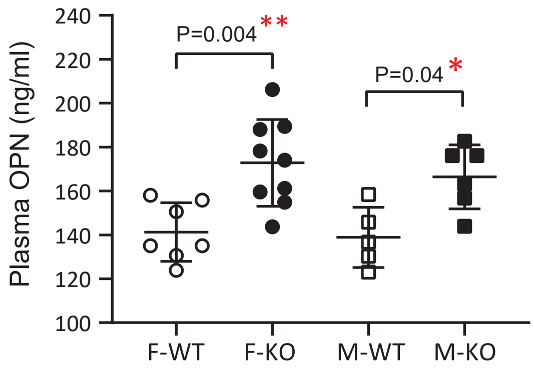

Fig. S8. KIAA1199 KO mice have higher plasma osteopontin (OPN) than corresponding wild-type (WT) mice in both female (F) and male (M). Plasma levels of OPN in KIAA1199 KO and WT mice were measured by ELISA. At least two independent animal experiments were performed, the representative results are shown here. Data are expressed as means ± SD, n (F-WT) = 7, n = (F-KO) = 10, n (M-WT) = 5, n (M-KO) = 6. Statistical difference was determined by one-way ANOVA with Tukey's multiple comparisons, *P < 0.05, **P < 0.01. Source data are provided as a Source Data file.

Supplementary Table 1. Sequences and gene names of 10 potential off-target sites in the mouse genome for KIAA1199 gRNA in this study

	Gene name	Sequence	Score	Mismatches	UCSC gene	Locus
1	Cacna2d2	TGTGTCCTCATTGATGACGGAGG	0.3	4MMs[2:3:4:9]	NM_001174047	Chr9:+107428239
2	Slc35g1	GGCATCCTGAGGGATGACGGGAG	0.4	4MMs[1:2:11:12]	NM_175507	Chr19:+38479679
3	Eda2r	TCCATCCTGGATGATGACTGCAG	0.3	4MMs[2:10:11:19]	NM_175540	ChrX:-94524386
4	Gcnt4	TACATTCTCCTTGATGACAGGAG	0.2	4MMs[6:9:10:19]	NM_001166065	Chr13:-97719810
5	Tnfaip8l3	AACATGCTGATTGATGACACCAG	0.1	4MMs[1:6:19:20]	NM_001033535	Chr9:-53875334
6	Macrod2	TGCATCCTGAATGATGGTGGAGG	0.1	4MMs[2:11:17:18]	NM_028387	Chr2:-140244531
7	Il31	TACATGCTGAGTGATGGGGGGGG	0	4MMs[6:11:17:18]	NM_029594	Chr5:-123930410
8	Fras1	GACATCCTGATAGGTGAAGGAGG	0	4MMs[1:12:14:18]	NM_175473	Chr5:-97138945
9	Tmem2	TACATCCTGATCCAGGATGGTGG	0	4MMs[12:13:15:18]	NM_001033759	Chr19:+21872414
10	Emsy	TACATCCTGATATATTATGGTGG	0	4MMs[12:13:16:18]	NM_172280	Chr7:+105751155

Supplementary Table 2 : PCR primers for 10 potential off-target genes in KIAA1199 knockout mice in the study

Gene ID	Gene name	Forward primer (5’ → 3’)	Reverse primer(5’ → 3’)
Cacna2d2	Calcium channel, voltage-dependent, alpha 2/delta subunit 2	ACCCTGACTCCCTCTTCTT	TCTTCCTGACCCTTACCTG
Slc35g1	Solute carrier family 35, member G1	TAAATCACCTTAGCCACC	CTTCCACTATCCCAGACA
Eda2r	Ectodysplasin A2 receptor	AAGACAGTGGAGGAGGATT	GGTATGGACCCATTGAGA
Gcnt4	Glucosaminyl (N-acetyl) transferase 4	CCTGTCATGGCATTTGGA	GGTCTGCCCATCATCCAC
Tnfaip8l3	Tumor necrosis factor, alpha-induced protein 8-like 3	ATGTTCCTGCTTGTATCTC	ATAACCTCCTCTTGACTG
Macrod2	MACRO domain containing 2	TAACCGACTCCCTTTCTT	CCCAACCTTTACTCCCTA
Il31	Interleukin 31	CTTGGGTGATAAGATGGC	TGGAGTTAAGTCCTGGGT
Fras1	Fraser extracellular matrix complex subunit 1	GTTGGCAAAGTTGGACTG	GAAATCACGAACCCATAAAA
Tmem2	Transmembrane protein 2	GGGACCCACATCTGCTAG	CCTCCACGCCAATAAACT
Emsy	EMSY, BRCA2-interacting transcriptional repressor	TCCCAGCCTGTAGTTCAT	CTTGTCCTGCATTTGTGC

Supplementary Table 3. PCR primers for different genes used in the Real time PCR in the study

Species	Gene ID	Gene name	Forward primer (5' → 3')	Reverse primer (5' → 3')	Length (bp)	Gene Sequence ID
Human	KIAA1199	Cell migration-inducing and hyaluronan-binding protein	TCTTTGGGCCACTGCTTCTTCACG	GTCTTGCCTGGGCTTGGGGATGTA	153	NM_001293298.1
Human	OPN	Osteopontin	CCAAGTAAGTCCAACGAAAG	GGTGATGTCCTCGTCTGTA	329	NM_000582
Human	ALP	Alkaline phosphatase, liver/bone/kidney (Alpl)	ACGTGGCTAAGAATGTCATC	CTGGTAGGCGATGTCCTTA	476	NM_001127501.4
Human	RUNX2	Runt-related transcription factor 2	TGGTTACTGTCATGGCGGGTA	TCTCAGATCGTTGAACCTTGCTA	101	NM_001015051.4
Human	GAPDH	Glyceraldehyde-3-phosphate dehydrogenase	GGCGATGCTGGCGCTGAGTAC	TGGTTCACACCCATGACGA	130	NM_002046
Human	B2M	Beta 2-microglobulin	CCTTGAGGCTATCCAGCGT	CCTGCTCAGATACATCAAACATG	510	NM_004048.2
Human	ACTB	Beta-actin	ATTGGCAATGAGCGGTTCCG	AGGGCAGTGATCTCCTTCTG	192	NM_001614.3
Mouse	KIAA1199	Cell migration-inducing and hyaluronan-binding protein	GAGAAAAGACAATGGGCATA	TTCCCATCAGACCCAACA	338	NM_030728
Mouse	Bglap	Bone gamma-carboxyglutamate protein (Osteocalcin precursor (OC, OCN))	TGCGCTCTGTCTCTCTGACC	CTGTGACATCCATACTTGCAGG	357	NM_001032298.3
Mouse	Col1a1	Collagen, type I, alpha 1	GGTGAACAGGGTGTTCCTGG	TTCGCACCAGGTTGGCCATC	503	NM_007742.4
Mouse	Alp	Alkaline phosphatase, liver/bone/kidney (Alpl)	GCCCTCTCCAAGACATATA	CCATGATCACGTCGATATCC	373	NM_007431.3
Mouse	Opn/SSP1	Osteopontin/secreted phosphoprotein 1	GAAACTCTTCCAAGCAATTC	GGACTAGCTTGTCCTTGTGG	589	NM_001204201.1
Mouse	Runx2	Runt-related transcription factor 2	AGCAACAGCAACAACAGCAG	GTAATCTGACTCTGTCCTTG	685	NM_001145920.2
Mouse	Opg	osteoprotegerin	CTTCTTCCCAGGCAGGCTCTCCAT	GGAACCCCAGAGCGAAACACAGT	519	NM_001411506.1
Mouse	Rankl	Receptor activator of nuclear factor kappa-B ligand	GGCCACAGCGCTTCTCAG	GAGTGACTTTATGGGAACCCGAT	144	NM_011613.4
Mouse	Rank/Tnfrsf11a	Tumor necrosis factor receptor superfamily member 11A	TGGGTGATTTTCTTTTGGTGGGT	TATGCCTGTGGCCTCTTTCAG	69	NM_009399.3
Mouse	Trap/Acp5	Tartrate-resistant acid phosphatase type 5	CTCTTGCAGCCTCTCTGACCA	CCATCGTCTGCACGGTTCT	228	NM_007388.3
Mouse	Ctsk	Cathepsin K	GTGGGCTCTTTCTGAGTTCTGT	CAGACACGAATGGAGGTGGAA	331	XM_006500974.4
Mouse	Crl/Calcr	Calcitonin receptor	CCTCTTGCCCTTGGGTGCTATC	CTGGGGAGTAAAGAGGGGTATGG	328	NM_001377018.1
Mouse	B2m	Beta 2-microglobulin	GCTATCCAGAAAACCCCTCAA	CATGTCTCGATCCCAGTAGACGGT	276	NM_009735.3
Mouse	Actb	Beta-actin	GATATCGCTGCGCTGGTCGTC	ACGCAGCTCATTGTAGAAGGTGTGG	217	NM_007393.5
